# Assessing Velocity and Directionality of Uterine Electrical Activity for Preterm Birth Prediction Using EHG Surface Records

**DOI:** 10.3390/s20247328

**Published:** 2020-12-20

**Authors:** Franc Jager, Ksenija Geršak, Paula Vouk, Žiga Pirnar, Andreja Trojner-Bregar, Miha Lučovnik, Ana Borovac

**Affiliations:** 1 Faculty of Computer and Information Science, University of Ljubljana, 1000 Ljubljana, Slovenia; paula.vouk@gmail.com (P.V.); Ziga.Pirnar@fri.uni-lj.si (Ž.P.); ab.borovac@gmail.com (A.B.); 2 Faculty of Medicine, University of Ljubljana, 1000 Ljubljana, Slovenia; ksenija.gersak@mf.uni-lj.si (K.G.); atb@t-2.net (A.T.-B.); mihalucovnik@yahoo.com (M.L.); 3 University Medical Center Ljubljana, 1000 Ljubljana, Slovenia

**Keywords:** electrohysterogram, propagation of EHG waves, short-time cross-correlation, dummy intervals, contraction intervals, conduction velocity, conduction velocity directionality, preterm birth prediction

## Abstract

The aim of the present study was to assess the capability of conduction velocity amplitudes and directions of propagation of electrohysterogram (EHG) waves to better distinguish between preterm and term EHG surface records. Using short-time cross-correlation between pairs of bipolar EHG signals (upper and lower, left and right), the conduction velocities and their directions were estimated using preterm and term EHG records of the publicly available Term–Preterm EHG DataSet with Tocogram (TPEHGT DS) and for different frequency bands below and above 1.0 Hz, where contractions and the influence of the maternal heart rate on the uterus, respectively, are expected. No significant or preferred continuous direction of propagation was found in any of the non-contraction (dummy) or contraction intervals; however, on average, a significantly lower percentage of velocity vectors was found in the vertical direction, and significantly higher in the horizontal direction, for preterm dummy intervals above 1.0 Hz. The newly defined features—the percentages of velocities in the vertical and horizontal directions, in combination with the sample entropy of the EHG signal recorded in the vertical direction, obtained from dummy intervals above 1.0 Hz—showed the highest classification accuracy of 86.8% (AUC=90.3%) in distinguishing between preterm and term EHG records of the TPEHGT DS.

## 1. Introduction

The World Health Organization (WHO) defines preterm birth, also referred to as premature birth or premature delivery, as live delivery of newborns that occurs before the 37th week of gestation [1]. The WHO reports the prevalence of preterm birth in 10% of newborns, or 15 million newborns every year.

The analysis of an electrohysterogram (EHG), i.e., uterine electromyogram (EMG), recorded from the abdominal wall of a pregnant woman is a promising, non-invasive, and low-cost technique for predicting preterm birth. Since the delivery is preceded by the effective uterine contractions, which are a result of the electrical activity within the myometrium due to the increased cell excitability and electrical coupling between myometrial cells, the analysis of the EHG surface records allows non-invasive monitoring of uterine dynamics and the extraction and analysis of different EHG parameters to distinguish between preterm and term pregnancies [2,3,4].

There are two major approaches to the task of predicting spontaneous preterm birth. The first approach tries to distinguish between the non-labor (pregnancy) phase and labor phase (either in preterm or term EHG records), while the second tries to distinguish between preterm and term EHG records [4,5,6]. Both classification approaches deal with individual contraction burst intervals (contractions, or contraction intervals), or with the entire set of EHG records or signals. Recently, individual non-contraction intervals (dummy intervals) of the EHG records were also successfully used to separate between preterm and term EHG records [5].

A large variety of linear and non-linear signal processing techniques have been used for predicting preterm birth. The investigated parameters (or features) for the analysis of the EHG signals and/or classification of preterm and term EHG records were temporal and spectral parameters; and for non-linear analysis, they were entropy parameters estimating regularity and predictability, as well as parameters estimating non-linearity and complexity [4,5]. The temporal parameters measuring the intensity of contraction intervals were: signal amplitude, area under contraction curve, and root mean square (RMS) value [7,8]. The spectral parameters estimating shifts and amplitude changes of the power spectrum during pregnancy were: peak, median, or dominant frequency of the power spectrum [9,10,11,12,13,14,15,16,17,18,19], normalized peak amplitude of the power spectrum [5], wavelets [13,20,21,22], and autoregressive coefficients [20,23]. Non-linear parameters estimate regularity, predictability, periodicity, the amount of chaos, and the complexity of a time series. A large number of parameters were investigated: sample entropy [5,6,9,12,13,14,15,16,17,18,19,24,25], variance entropy [13], approximate entropy [6,26], time reversibility [6,13,14,25,26], multivariate multiscale fuzzy entropy [27], spectral entropy [25,28], entropy of intrinsic mode functions [29], fractal dimension, interquartile range, mean Teager–Kaiser energy [24], and Lempel–Ziv index complexity measure [25,30].

Using the EHG records of the publicly available Term–Preterm EHG DataBase (TPEHG DB) [9,31], several complex machine learning techniques were applied, and several excellent classification accuracies (up to 100%) in terms of classifying between preterm and term EHG records were obtained [5,8,15,16,17,18,19,23,24,25,27,29]. However, in the research community of this area, it has been well known that such high classification performances are likely over-estimated. These excellent results may be biased due to over-learning (no “hidden” set of records is available for testing) and due to an extremely unbalanced database (38 preterm vs. 262 term records), which requires balancing the number of examples in both classes.

Despite the large number of investigated parameters, extensive research has verified complex machine learning techniques; due especially to the relatively small number of representative examples in currently available EHG databases, it is still not quite clear what would be the “best” features for which to maximize the classification accuracy as much as possible for efficient classification between preterm and term EHG records.

In addition to the temporal, spectral, and non-linear parameters already discussed, the EHG electrical activity, as measured on the abdominal wall, also allows propagation analysis of EHG signals [32], propagation analysis of uterine contractions [33], and evaluation of directionality and synchronization of the EHG signals [34]. These include: estimation of conduction or propagation velocity amplitudes [10,35,36,37,38,39,40,41,42], estimation of conduction velocity directions [39,40,41,42,43], estimation of propagation patterns [40,42], estimation of directionality, synchronization, and coupling [34,44], and estimation of spatiotemporal EHG patterns [45]. From now on, we will only focus on the estimation of conduction velocity amplitudes and directions.

For the analysis of the propagation of the EHG signals, a variety of unipolar or bipolar electrode configurations measuring the EHG action potentials, or the EHG signals, were used: 64 unipolar signals (from a grid of 8 × 8 high-density electrodes) [35,36,38,42], 16 unipolar signals (from a grid of 4 × 4 electrodes) [41], four unipolar signals [43], three bipolar signals [39], or two bipolar (from two electrode pairs, upper and lower) signals [10]. In order to estimate conduction velocity amplitude or directionality, different signal processing techniques were used: maximum likelihood approach [35,41], maximum likelihood approach and sliding window [36,38], calculating the time difference between the centers of mass of the EHG bursts [39], calculating inter-correlation functions between the EHG signals [33], inter-channel correlation by means of the Pearson product-moment correlation coefficient between the vertical and horizontal pairs of two adjacent electrodes [42], running cross-correlation windowing [43], and visual identification [10]. Moreover, in these studies, the following frequency bands of the EHG signals used were: 0.1–1.0 Hz [41], 0.3–1.0 Hz [35], 0.34–1.0 Hz [10,39], 0.1–0.8 Hz [36], 0.3–0.8 Hz [38], and 0.35–0.8 Hz [42]. These frequency bands were selected because the goal was to estimate conduction velocity amplitude or directionality within uterine contractions. Only two studies based on estimating the conduction velocity amplitude or directionality also differentiated between the non-labor and labor contractions (including true preterm cases) [10,38], but none of the studies differentiated between preterm and term EHG surface records.

The purpose of this study was to assess the capabilities of conduction velocity amplitudes and directions of propagation of the EHG waves using the publicly available Term–Preterm EHG DataSet with Tocogram (TPEHGT DS) [5,31], which contains human-annotated dummy and contraction intervals to better distinguish between preterm and term EHG surface records. Newly defined individual features, the percentages and average conduction velocities in the vertical and horizontal directions, and their ratios for dummy and contraction intervals were investigated. Our previous study [5] showed that the excitability of the uterus is not restrained to efficient contractile events, which represent only a small fraction of the total duration of pregnancy, but important physiological mechanisms are also present during dummy intervals, and at higher frequencies above 1.0 Hz. Therefore, in this study, both, dummy and contraction intervals were investigated in separate frequency bands below and above 1.0 Hz.

The main aims of this study were:

1. To characterize dummy and contraction intervals of the EHG records of the publicly available TPEHGT DS in terms of conduction velocity amplitudes and conduction velocity directions of the EHG surface electric potential;
2. To test the hypothesis that the newly defined features, the percentages and average conduction velocities in the vertical and horizontal direction derived from the frequency bands above 1.0 Hz (influenced by the maternal heart rate with higher harmonics), are important for the efficient prediction of preterm birth, and possibly to identify new and simple biophysical markers;
3. To confirm the hypothesis [5] that dummy intervals of the EHG records are equally—or even more—as important for the accurate prediction of preterm birth as contraction intervals are;
4. To compare the individual classification performance of the newly defined features with the performances of the previously established sample entropy features of the original EHG signals.

In this study, the short-time cross-correlation technique was used to estimate the conduction velocities and directions of propagation of the EHG waves. The technique was applied over entire dummy and contraction intervals, and over entire EHG records, using a variety of selected frequency bands below and above 1.0 Hz. Below 1.0 Hz, mainly the physiological mechanism of contractions is expected, while above 1.0 Hz, the influence of the maternal heart rate on the uterus is expected. Characterization of dummy and contraction intervals revealed no obvious or preferred continuous direction of propagation, but on average, a significantly lower percentage of velocity vectors in the vertical direction and a significantly higher percentage of velocity vectors in the horizontal direction were found for preterm dummy intervals in the frequency bands above 1.0 Hz. The lower number of EHG propagation waves in the vertical direction means a reduced influence of the maternal heart on the uterus in the vertical direction for preterm pregnancies. This is an important new marker for assessing the risk of preterm birth. The individual classification accuracies of these newly defined individual features and of pairs of features obtained from dummy intervals of records in the frequency region above 1.0 Hz yielded higher classification accuracies in distinguishing between preterm and term records of the TPEHGT DS than these individual features or pairs of features obtained from contraction intervals of the records.

## 2. Materials and Methods

### 2.1. Dataset

**Figure 1:**
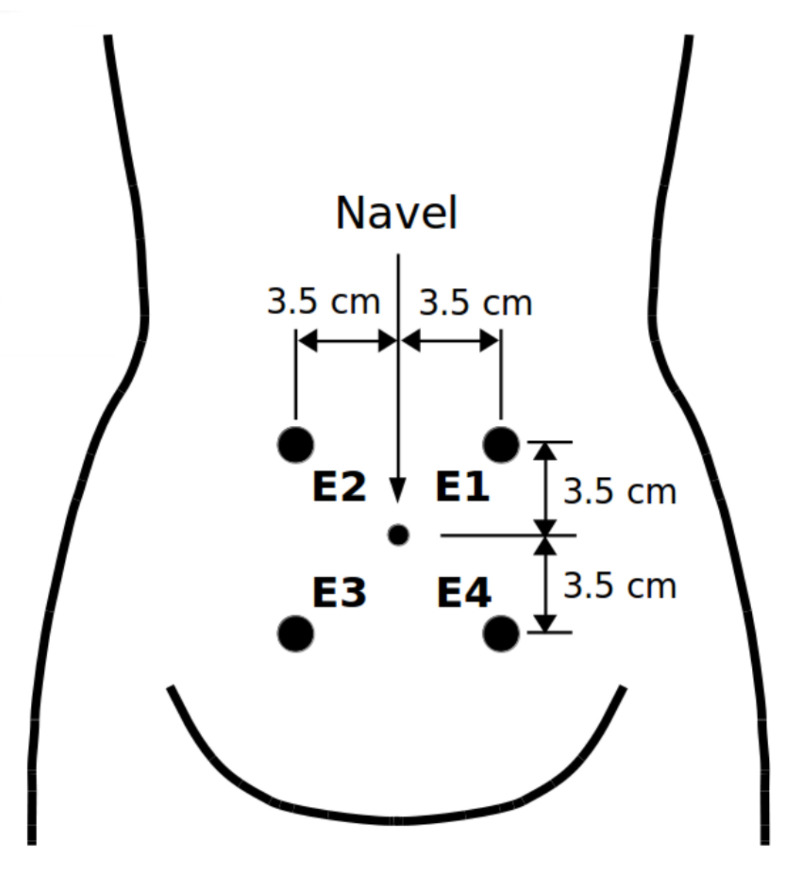
Positions of electrodes. The electrodes were placed symmetrically at a distance of 7 cm in two horizontal rows, above and under the navel [5].

The publicly available TPEHGT DS [5,31] used in this study contains 13 uterine EHG records that resulted in spontaneous preterm delivery, 13 uterine EHG records that resulted in spontaneous term delivery, and another five uterine EHG records of non-pregnant women. Each record is 30 min in length and contains three EHG signals and a simultaneously recorded TOCO signal (external tocogram, measuring the external mechanical uterine pressure). The records were collected at the University Clinical Centre Ljubljana, Gynaecology Clinic, Division of Perinatology. The mean delivery times of preterm and term records were 33.7 ± 1.97 and 38.1 ± 1.04 weeks of pregnancy, while the mean recording time of the records of pregnant women was 30.2 ± 2.76 weeks. The positions of electrodes to measure the EHG signals are shown in Figure 1.

**Figure 2:**
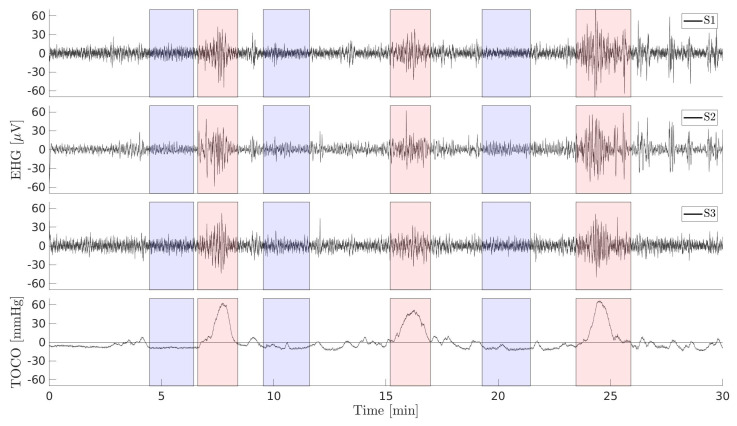
The electrohysterogram (EHG) and external tocogram (TOCO) signals of the record *tpehgt_p008* (preterm, delivery in the 32nd week, recorded in the 26th week of pregnancy). Blue: human-annotated dummy intervals, red: human-annotated contraction intervals.

The EHG signals of the records were acquired using AgCl${}_{2}$ electrodes from the abdominal wall. The first bipolar EHG signal (S1) was measured between the two top electrodes (E2 − E1), the second (S2) between the two left electrodes (E2 − E3), and the third (S3) between the two lower electrodes (E4 − E3). The analog EHG signals were filtered using an analog three-pole Butterworth filter with the bandwidth 0.0–5.0 Hz. The sampling frequency, $f_{S}$, was 20 Hz. The records of the dataset contain 47 human-annotated contraction intervals related to uterine contractions and 47 human-annotated dummy intervals in preterm records, as well as 53 human-annotated contraction and 53 human-annotated dummy intervals in term records. The average lengths of preterm and term contraction intervals are 82 ± 48 s and 88 ± 36 s, and for preterm and term dummy intervals, they are 83 ± 46 s and 89 ± 46 s. The EHG signals, TOCO signals, and manually annotated contraction and dummy intervals of a preterm record of the TPEHGT DS are shown in Figure 2.

### 2.2. Physiological Mechanisms and Selected Frequency Bands

EHG signals carry information about the underlying physiological mechanisms of the uterus present during pregnancy. These mechanisms are non-linear processes; therefore, EHG signals vary over time and are non-stationary. Activity below 0.3 Hz is expected due to maternal breathing [46]; therefore, this region needs to be excluded. The main activity connected to the mechanism responsible for uterine contractions is expected in the frequency band 0.3–1.0 Hz [10,11,15,17,18]. It was also estimated that the main EHG spectral content of the uterine bursts distributes up to 4.0 Hz [47,48]. Moreover, above 1.0 Hz, especially during dummy intervals, the frequency component of the maternal heart rate (maternal ECG) and its higher harmonics are expected. The physiological mechanism related to the maternal heart resides in separate frequency bands, but their frequency content and intensities significantly vary within the bands as the pregnancy progresses [5]. Therefore, in this study, we characterized conduction velocity amplitudes and directions in the following different and separate frequency bands:

- Band B0${}^{\prime }$: $\;\;f_{\mathrm{low}}$ = 0.3 Hz, $\;\;\;f_{\mathrm{high}}$ = 1.0 Hz;
- Band Bb: $\;\;\;f_{\mathrm{low}}$ = 0.3 Hz, $\;\;\;f_{\mathrm{high}}$ = 4.0 Hz.
- Band B1: $\;\;\;f_{\mathrm{low}}$ = 1.0 Hz, $\;\;\;f_{\mathrm{high}}$ = 2.2 Hz;
- Band B2: $\;\;\;f_{\mathrm{low}}$ = 2.2 Hz, $\;\;\;f_{\mathrm{high}}$ = 3.5 Hz;
- Band B3: $\;\;\;f_{\mathrm{low}}$ = 3.5 Hz, $\;\;\;f_{\mathrm{high}}$ = 5.0 Hz;

### 2.3. Cross-Correlation, Calculation of the Fourth EHG Signal, and Preprocessing

The fundamental technique of this study is cross-correlation between two signals. Cross-correlation, $C_{x,y}\left(m\right)$, between two signals *x* and *y* is a procedure that identifies similarity between these two signals, or is a measure of similarity of these two signals for different time shifts, *m*, or lags [49],

(1) $$C_{x,y}\left(m\right)\;\;=\sum_{m=-\infty }^{\infty }x\left(n\right)\;y\left(n-m\right)\;.$$

**Figure 3:**
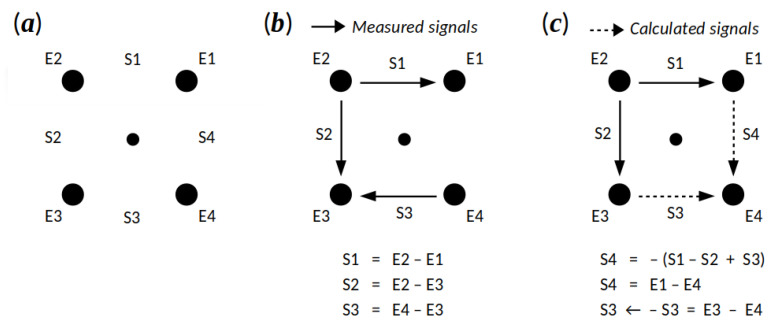
(**a**) Positions of the electrodes measuring the EHG potentials on the abdominal surface; (**b**) measured EHG signals of the records of the Term–Preterm EHG DataSet with Tocogram (TPEHGT DS) and their orientations. (**c**) Calculated and consistently oriented EHG signals.

In order to estimate the conduction velocity of an EHG wave and its direction on the surface of the abdomen, i.e., in two dimensions, using the EHG signals and cross-correlation, at least four EHG signals are needed, as measured by at least four electrodes (refer to Figure 3a). Cross-correlation between the upper horizontal signal S1 (the difference in electric potentials between electrodes E2 and E1) and the lower horizontal signal S3 (potential difference between E3 and E4), $C_{S1,S3}\left(m\right)$, will yield an estimate of similarity, or similarity shift, $s_{S1,S3}$, between the horizontal signals S1 and S3. This shift, $s_{S1,S3}$, is defined by the position of the maximum of $C_{S1,S3}\left(m\right)$. Cross-correlation between the left vertical signal S2 (potential difference between E2 and E3) and the right vertical signal S4 (potential difference between E1 and E4), $C_{S2,S4}\left(m\right)$, will yield an estimate of similarity, or similarity shift, $s_{S2,S4}$, between the vertical signals S2 and S4, defined by the position of the maximum of $C_{S2,S4}\left(m\right)$. These two shifts then allow the calculation of the conduction velocity amplitude and direction of propagation of an EHG wave (described in the following subsections).

The records of the TPEHGT DS provide only three EHG signals, S1, S2, and S3 (see Figure 3b). Therefore, the fourth signal, S4, needed to be derived. With regard to the positions of the electrodes and the pattern of orientations of the three signals available, the fourth signal S4 can be calculated as the difference of the EHG potentials of the electrodes E4 and E1. Since S1=E2−E1, S2=E2−E3, and S3=E4−E3, and using E4=S3+E3 and E1=E2−S1, it follows that:

(2) S4=E4−E1=S3+E3−E2+S1=S1−S2+S3.

The signals of the horizontal pair (S1 and S3) and the signals of vertical pair (S2 and S4) need to be equally oriented for the proper calculation of cross-correlation. Therefore, multiplication of the signals S3 and S4 by −1 follows (Figure 3c). For the calculation of the signal S4, the signals S1, S2, and S3 of the records were initially filtered using the fourth-order band-pass digital Butterworth filter, with cut-off frequencies at 0.08 and 5.0 Hz, which was applied bi-directionally to yield zero-phase shift. The Butterworth filters also provide monotonically changing amplitude response with no ripples in passband and stopband, which rolls off after bi-directional use at −160 dB per decade (the signals S1, S2, and S3 are, as such, readily available in the TPEHGT DS). After that, all four signals of the records were preprocessed using the same Butterworth filter and the same filtering technique, but according to the low and high frequencies of the selected frequency bands B0${}^{\prime }$, Bb, B1, B2, and B3.

**Figure A1:**
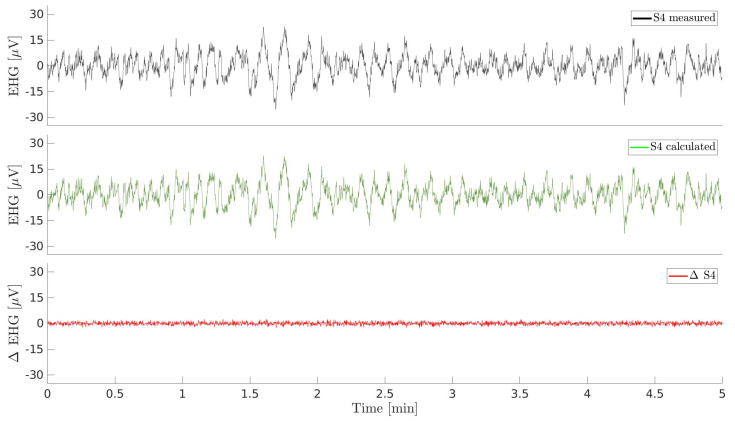
A five-minute excerpt of an originally recorded EHG signal $S4_{M}$ (upper trace), calculated EHG signal $S4_{C}$ (middle trace), and the difference between samples of signals, ΔS4 (lower trace), of a selected term EHG record. The standard deviation (Equation (A1)) for this entire record equals 0.78 (μV)${}^{2}$.

We verified discrepancies between actually measured and synthetically derived signal S4. A few EHG records were used for which the signal S4 was actually recorded (the signal S4 was recorded only for a few EHG records). The discrepancies between the calculated and actually measured signals were estimated using the standard deviation between the samples of signals, and are summarized in Appendix A. The discrepancies are negligible. An example is shown in Figure A1.

### 2.4. Short-Time Cross-Correlation

In this study, the conduction velocities and directions of propagation of the EHG waves were calculated over entire records using short-time cross-correlation. Short-time cross-correlation between two signals is a procedure where two simultaneous signals are divided into short, consecutive time segments of equal length, and then cross-correlation between the two signals (within a correlation window that is of finite duration and can be wider than the length of consecutive time segments) is applied at the center of each of these short, consecutive time segments. Short-time cross-correlation can also be applied using overlapping time segments, or using a running cross-correlation window at each signal sample. Such a short time segment will be referred to as a correlation interval. In our case, short-time cross-correlation was calculated for each pair of signals ((S1, S3) and (S2, S4)) of the records, resulting in two sequences of similarity shifts, $s_{S1,S3}\left(k\right)$, and $s_{S2,S4}\left(k\right)$, where *k* denotes the index of the correlation interval. The duration of the correlation window, $w_{C}$, was set to 5 s, and the length of the consecutive correlation intervals, $d_{\mathrm{CI}}$, to 0.5 s, while the interval within which the maxima of $C_{S1,S3}\left(k\right)$ and $C_{S2,S4}\left(k\right)$—defining the similarity shifts $s_{S1,S3}\left(k\right)$ and $s_{S2,S4}\left(k\right)$ between the pairs of signals—were searched for, $d_{\mathrm{SS}}$, was set to ± 1.0 s. Prior to the calculation of cross-correlations at the *k*-th correlation interval, the signals within the correlation window, $w_{C}$, were normalized. Each normalized signal, $S_{N}$, was calculated using:

(3) $$S_{N}\;\;=\;\;\frac{S-\min(S)}{\max(S-\min(S))}\;.$$

According to the order of the correlated signals (Equation (1)), the similarity shifts tell us about the direction of propagation of the EHG waves. The directions of propagation of the EHG waves can be estimated for all four possible plane directions. Positive similarity shift $s_{S1,S3}\left(k\right)$ means that the EHG wave travelled upwards at the *k*-th correlation interval, while negative shift means that it travelled downwards. Accordingly, positive similarity shift $s_{S2,S4}\left(k\right)$ means traveling to the right, while negative means traveling to the left. For these reasons, the similarity shift $s_{S1,S3}\left(k\right)$ can also be referred to as the vertical shift, $s_{V}\left(k\right)=s_{S1,S3}\left(k\right)$, and $s_{S2,S4}\left(k\right)$ as the horizontal shift, $s_{H}\left(k\right)=s_{S2,S4}\left(k\right)$.

### 2.5. Estimating Conduction Velocity Amplitudes and Directions of Propagation of EHG Waves

The spreading of the EHG electric potentials appears in waves. The source of propagating of EHG waves is not known, but it is possible to measure the changes of the EHG electric potentials on the abdominal surface. The velocities and directions of propagation of the EHG waves were estimated using the EHG signals recorded from four electrodes forming a square on the surface of the abdomen (Figure 1). Since the EHG electric potentials are measured from the abdominal surface in two dimensions, we assume EHG waves as planar waves. The origins, directions, and amplitudes of consecutive EHG waves are not known, and vary over time. In this study, we assume that the propagation is linear during each consecutive *k*-th correlation interval, thus allowing step-by-step robust exploitation and estimation of the velocities and directions. Using short-time cross-correlation between the pairs of signals, the vertical and horizontal shifts between the pairs of signals are known for each correlation interval.

**Figure 4:**
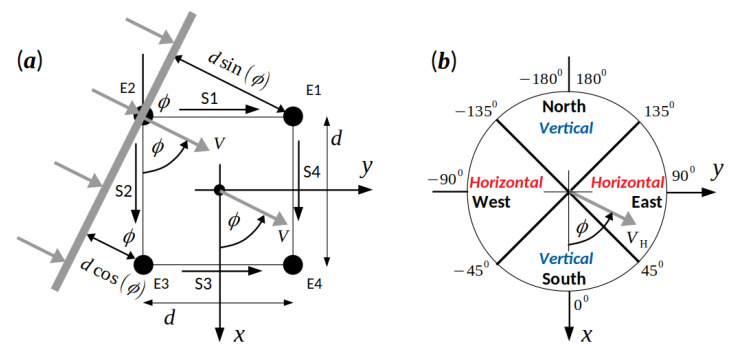
(**a**) Schematic description of the method used to estimate conduction velocity amplitudes and directions of EHG waves (adopted from [36]). (**b**) Distribution of sectors (South, East, North, and West) and propagation directions (Vertical and Horizontal) on the abdominal wall according to the selected coordinate system.

Figure 4a shows a schematic description of the method used to estimate the conduction velocity amplitudes and directions of the EHG waves [36]. Consider a linear EHG wave represented by a velocity vector *V* that will pass the electrodes on the abdominal surface, with a conduction velocity amplitude CV, and with an incidence angle ϕ. The incidence angle ϕ is defined between the vertical *x* axis of the selected coordinate system and the velocity vector *V*.

**Figure A2:**
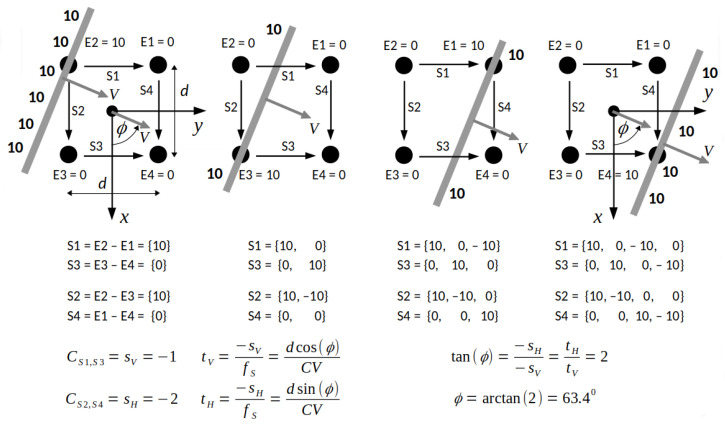
A detailed example of passing an EHG wave with an amplitude of, e.g., 10 μV, over the electrodes placed on the abdominal wall, the values of the corresponding EHG signals, the cross-correlation similarity shifts, $s_{V}=C_{S1,S3}$ and $s_{H}=C_{S2,S4}$, the transition times, $t_{V}$ and $t_{H}$, and the calculated incidence angle ϕ.

After leaving the electrode E2, the wave further propagates in the direction of velocity vector *V*. The distance to be passed by the wave in order to reach the electrode E3 is dcos(ϕ). Since the electrodes E2 and E3 are located on the same vertical line, and according to geometry, this distance is also the vertical distance, $d_{V}$, passed by the wave. Similarly, the distance to be passed by the wave, in order to reach the electrode E1, is dsin(ϕ). Since the electrodes E2 and E1 lie on the same horizontal line, this distance is also the horizontal distance, $d_{H}$, passed by the wave. To calculate the conduction velocity amplitude CV of the wave, the transitional time, $t_{V}$, needed to pass the vertical distance, $d_{V}$, and the transitional time, $t_{H}$, needed to pass the horizontal distance, $d_{H}$, have to be estimated. While the wave was passing the electrodes, the values of signals S1, S2, S3, and S4 changed accordingly. The vertical and horizontal transitional times, $t_{V}$ and $t_{H}$, are determined by the similarity shifts, $s_{V}$ and $s_{H}$, which are the result of cross-correlations, $C_{S1,S3}$ and $C_{S2,S4}$, at the *k*-th correlation interval, respectively:

(4) $$t_{V}\;\;=\;\;\frac{-s_{V}}{f_{S}}\;,$$

(5) $$t_{H}\;\;=\;\;\frac{-s_{H}}{f_{S}}\;,$$

where $f_{S}$ is the sampling frequency. Note the multiplication with −1 in both equations. In Equation (4), this is necessary due to the orientation of the signals S1 and S3, while in Equation (5), this is due to the selected coordinate system (see Figure 4a). A detailed synthetic example of passing an EHG wave over the electrodes, calculating cross-correlations, calculating the similarity shifts, $s_{V}$ and $s_{H}$, and the transitional times, $t_{V}$ and $t_{H}$, and calculating the incidence angle ϕ is provided in Figure A2 in Appendix B.

The transitional times, $t_{V}$ and $t_{H}$, and the conduction velocity amplitude, CV, are related according to [36]:

(6) $$t_{V}\;\;=\;\;\frac{d_{V}}{CV}\;\;=\;\;\frac{d\cos(\phi )}{CV}\;,$$

(7) $$t_{H}\;\;=\;\;\frac{d_{H}}{CV}\;\;=\;\;\frac{d\sin(\phi )}{CV}\;.$$

Since:

(8) $$t_{V}^{2}\;+\;t_{H}^{2}\;\;=\;\;\frac{d^{2}\sin^{2}\left(\phi \right)}{CV^{2}}\;+\;\frac{d^{2}\cos^{2}\left(\phi \right)}{CV^{2}}\;\;=\;\;\frac{d^{2}}{CV^{2}}\;,$$

the CV follows:

(9) $$CV\;\;=\;\;\frac{d}{\sqrt{t_{V}^{2}\;\;+\;\;t_{H}^{2}}}\;.$$

Similarly, using $t_{V}$ and $t_{H}$, the incidence angle ϕ of the velocity vector *V* follows:

(10) $$\tan\left(\phi \right)\;\;=\;\;\frac{t_{H}}{t_{V}}\;\;=\;\;\frac{d_{H}}{d_{V}}\;\;=\;\;\frac{\sin(\phi )}{\cos(\phi )}\;,$$

(11) $$\phi \;\;=\;\;\arctan(\frac{t_{H}}{t_{V}})\;.$$

The conduction velocity amplitude CV and direction of the incidence angle ϕ of the velocity vector *V* were estimated for each *k*-th correlation interval. To calculate them, only the vertical and horizontal transitional times, $s_{V}$ and $s_{H}$, are needed. If both transitional times equal zero, it means that there was no propagation. For these correlation intervals, the CV was set to zero, and ϕ was marked as undefined. The cases where the calculated CV values were above 30 cm/s, thus significantly higher than the physiological values reported in the literature [50], were considered as outliers [38]. For extremely high conduction velocities, the estimated transitional times are extremely short. We assume that this was due to EHG activities with no propagation, or due to malfunction of the estimation method. Since the estimated transitional times were extremely short and close to zero, for these correlation intervals, the CV was also set to zero and ϕ was marked as undefined.

If propagation of the EHG waves was present for the *k*-th correlation interval, i.e., CV≠0, then the corresponding velocity vector *V* was aligned into one of the propagation directions (vertical and horizontal) and into one of the sectors (north, east, south, and west) according to the incidence angle ϕ of the velocity vector *V*. Figure 4b shows the distribution of the sectors and propagation directions. The alignment rules are the following:

- If $\left({-45}^{\circ }\leq \phi <45^{\circ }\right)$, then $\;\;\;\;V\to V_{V}\;\;\;$(vertical), $\;\;\;\;\;\;\;\;\;\;\;V\to V_{S}\;\;\;\;$(south).
- If $\left(45^{\circ }\leq \phi <135^{\circ }\right)$, then $\;\;\;\;V\to V_{H}\;\;\;$(horizontal), $\;\;\;V\to V_{E}\;\;\;\;$(east).
- If $\left(135^{\circ }\leq \phi <{-135}^{\circ }\right)$, then $\;\;\;\;V\to V_{V}\;\;\;$(vertical), $\;\;\;\;\;\;\;\;\;\;\;V\to V_{N}\;\;\;$(north).
- If $\left({-135}^{\circ }\leq \phi <{-45}^{\circ }\right)$, then $\;\;\;\;V\to V_{H}\;\;$(horizontal), $\;\;\;\;V\to V_{W}\;\;$(west).

### 2.6. Selected Parameters for Characterization of EHG Records

The conduction velocity amplitudes and directions of propagation of the EHG electrical activity were estimated for all correlation intervals over each entire EHG record of the TPEHGT DS. Initially, average conduction velocities, $\bar{CV}$, were calculated for dummy and contraction intervals in each record and for each entire record using the estimated conduction velocities in the correlation intervals. After that, the percentages, $P_{V}$ and $P_{H}$, as well as $P_{S}$, $P_{E}$, $P_{N}$, $P_{W}$, and the average conduction velocities, ${\bar{V}}_{V}$ and ${\bar{V}}_{H}$, as well as ${\bar{V}}_{S}$, ${\bar{V}}_{E}$, ${\bar{V}}_{N}$, ${\bar{V}}_{W}$, of velocity vectors *V* according to direction (vertical or horizontal) and according to sector (south, east, north, and west), respectively, were calculated for each dummy and each contraction interval of the records. This time, to calculate the average conduction velocities, ${\bar{V}}_{V}$ and ${\bar{V}}_{H}$, as well as ${\bar{V}}_{S}$, ${\bar{V}}_{E}$, ${\bar{V}}_{N}$, ${\bar{V}}_{W}$), the correlation intervals with CV=0 and undefined incidence angle ϕ were excluded. If CV=0 and ϕ is undefined, the direction and sector are unknown. In addition, the ratios between the vertical and horizontal percentages, $RP_{\mathrm{VH}}$, and ratios between the vertical and horizontal average conduction velocities, $RV_{\mathrm{VH}}$, were calculated. All these parameters were calculated for each of the investigated frequency bands: B0${}^{\prime }$, Bb, B1, B2, and B3, and will be referred to as the EHG propagation features.

### 2.7. Selected Features for Classification

The selected EHG propagation features to separate between preterm and term EHG records using dummy or contraction intervals were:

1. Percentage of correlation intervals with velocity vectors in the vertical direction:
  (12) $$P_{V}\;\;=\;\;\frac{N_{V}}{N}\cdot 100\;,$$
  where $N_{V}$ is the number of correlation intervals with velocity vectors *V* of which CV≠0 in the vertical direction, and *N* is the total number of correlation intervals.
2. Percentage of correlation intervals with velocity vectors in the horizontal direction:
  (13) $$P_{H}\;\;=\;\;\frac{N_{H}}{N}\cdot 100\;,$$
  where $N_{H}$ is the number of correlation intervals with velocity vectors *V* of which CV≠0 in the horizontal direction.
3. Ratio of percentages of correlation intervals with velocity vectors in the vertical and horizontal direction:
  (14) $$RP_{\mathrm{VH}}\;\;=\;\;\frac{P_{V}}{P_{H}}\;.$$
4. Average conduction velocity in the vertical direction:
  (15) $${\bar{V}}_{V}\;\;=\;\;\frac{1}{N_{V}}\;\;\sum_{i=1}^{N_{V}}\;\;CV\left(i\right)\;,$$
  where CV(i) are conduction velocities of correlation intervals with velocity vectors *V* in the vertical direction, CV(i)≠0, and $N_{V}$ is the number of velocity vectors *V* in the vertical direction.
5. Average conduction velocity in the horizontal direction:
  (16) $${\bar{V}}_{H}\;\;=\;\;\frac{1}{N_{H}}\;\;\sum_{i=1}^{N_{H}}\;\;CV\left(i\right)\;,$$
  where CV(i) are conduction velocities of correlation intervals with velocity vectors *V* in the horizontal direction, CV(i)≠0, and $N_{H}$ is the number of velocity vectors *V* in the horizontal direction.
6. Ratio of average conduction velocities in the vertical and horizontal directions:
  (17) $$RV_{\mathrm{VH}}\;\;=\;\;\frac{{\bar{V}}_{V}}{{\bar{V}}_{H}}\;.$$

The separation and classification abilities of these EHG propagation features were compared to the abilities of the signal sample entropy [51,52] feature. This feature has already proven its high classification power in classifying preterm and term EHG records to predict preterm birth [5,12,15,16,17,18,19,24,25]. Sample entropies of the original EHG signals S2, $SE_{S2}$, and S3, $SE_{S3}$, were used. The $SE_{S2}$ estimates regularity of the EHG signal in the vertical (S2) direction, while the $SE_{S3}$ estimates in the horizontal (S3) direction. Sample entropy requires two parameters: the length of matching patterns, *m*, and the matching margin, *r*. The values for these parameters, m=3 and r=0.15, were adopted from a previous study [9].

### 2.8. Assessing Separability, Classification, and Performance Measures

To estimate the ability of individual selected features to separate between preterm and term dummy and contraction intervals, a two-sample *t*-test with a pooled variance estimate was used [53]. The classifier used in this study was the QDA classifier. This classifier has already reliably classified between the labor and pregnancy contractions [13], between preterm and term records of the TPEHG DB [5,19], and between dummy and contraction intervals of the TPEHGT DS [5,54].

Due to the unequal number of samples (47 preterm versus 53 term dummy and contraction, intervals) in the TPEHGT DS, the standard synthetic minority over-sampling technique (SMOTE) [55] was employed to balance both classes using the over-sampling approach, thus forcing the decision region of the minority class to become more general.

Classification performances were assessed in terms of sensitivity, Se=TP/(TP+FN), specificity, Sp=TN/(TN+FP), classification accuracy, CA=(TP+TN)/(TP+FN+TN+FP), and AUC (area under the receiver operating characteristic curve) [56] (TP denotes the number of true positives, TN the number of true negatives, FN the number of false negatives, and FP the number of false positives). A cross-validation technique with 10 folds and with 30 repetitions was used in each case.

## 3. Results

### 3.1. Average Conduction Velocity Amplitudes of Preterm and Term Dummy and Contraction Intervals

**Figure A3:**
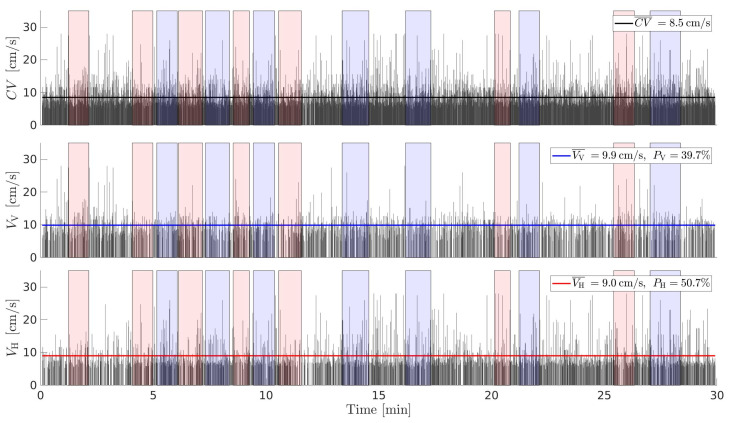
Conduction velocity amplitudes, CV, and conduction velocity amplitudes in the vertical, $V_{V}$, and horizontal, $V_{H}$, directions of the record *tpehgt_p006* in the frequency band B1. $\bar{CV}$—the average conduction velocity. ${\bar{V}}_{V}$, ${\bar{V}}_{H}$—the average conduction velocities (CV≠0) in the vertical and horizontal directions. $P_{V}$, $P_{H}$—the percentages of conduction velocities in the vertical and horizontal directions. Blue: dummy intervals, red: contraction intervals.

**Figure A4:**
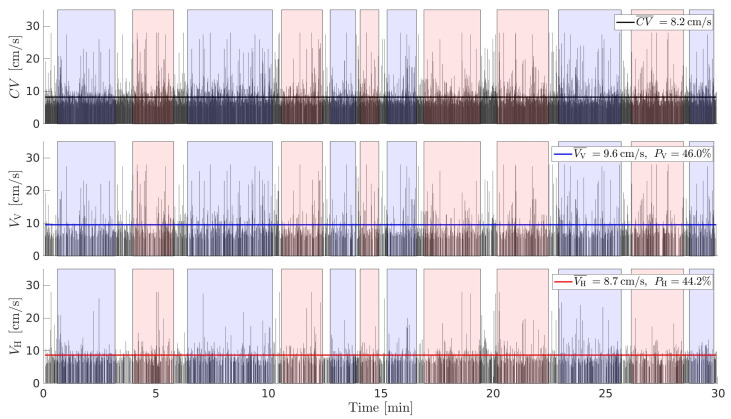
Conduction velocity amplitudes, CV, and conduction velocity amplitudes in the vertical, $V_{V}$, and horizontal, $V_{H}$, directions of the record *tpehgt_t009* in the frequency band B1. $\bar{CV}$—the average conduction velocity. ${\bar{V}}_{V}$, ${\bar{V}}_{H}$—the average conduction velocities (CV≠0) in the vertical and horizontal directions. $P_{V}$, $P_{H}$—the percentages of conduction velocities in the vertical and horizontal directions. Blue: dummy intervals, red: contraction intervals.

Table 1 shows the aggregate average conduction velocity amplitudes, $\bar{CV}$, as estimated from preterm and term dummy and contraction intervals, and from the entire set of EHG records of the TPEHGT DS in the frequency bands B0${}^{\prime }$, Bb, B1, B2, and B3. They do not differ significantly between preterm and term records. In the frequency band B1, the aggregate average $\bar{CV}$ is practically equal for preterm and term dummy intervals (8.42 ± 0.55 cm/s vs. 8.45 ± 0.73 cm/s), but is slightly lower for preterm contraction intervals (8.03 ± 0.75 cm/s vs. 8.31 ± 0.60 cm/s). We may conclude that the conduction velocity amplitudes are a bit higher for preterm records in the bands B0${}^{\prime }$ and Bb, where mainly electrical activity due to contractions is expected, and a bit lower in the bands B1, B2, and B3, where mainly electrical activity due to the influence of the maternal heart on the uterus is expected. Appendix C summarizes the average conduction velocity amplitudes, $\bar{CV}$, as estimated from each individual record for preterm and term dummy and contraction intervals (Table A1), and from each entire EHG record (Table A2). Time courses of the conduction velocity amplitudes, CV, and conduction velocity amplitudes in the vertical, $V_{V}$, and horizontal, $V_{H}$, directions for a preterm (*tpehgt_p006*; Figure A3) and for a term (*tpehgt_t009*; Figure A4) EHG record in the frequency band B1 are also provided.

### 3.2. Percentages and Average Conduction Velocities in the Vertical and Horizontal Directions

Table 2 summarizes the percentages of correlation intervals with the velocity vector in the vertical, $P_{V}$, and horizontal, $P_{H}$, directions, their ratios, $RP_{\mathrm{VH}}$, the average conduction velocity amplitudes in the vertical, ${\bar{V}}_{V}$, and horizontal, ${\bar{V}}_{H}$, directions, and their ratios, $RV_{\mathrm{VH}}$, for dummy and contraction intervals of preterm and term records of the TPEHGT DS and for each of the selected frequency bands, B0${}^{\prime }$, Bb, B1, B2, and B3. Recall that when dealing with the directions and sectors, all correlation intervals with CV=0 are excluded from the analysis.

In the frequency band B0${}^{\prime }$, where contractions are expected, the higher percentages of the vertical velocity vectors, $P_{V}$, are present for both types of intervals and for both types of records, while in the frequency bands Bb, B1, B2, and B3, the percentages of horizontal velocity vectors, $P_{H}$, prevail (with dummy intervals of term records being the exception). Much higher percentages of horizontal velocity vectors, $P_{H}$, and much lower percentages of vertical velocity vectors, $P_{V}$, are present for dummy intervals of preterm records in the frequency bands Bb (35.9% vs. 29.7%), B1 (45.2% vs. 32.7%), B2 (45.4% vs. 36.5%), and B3 (49.4% vs. 37.2%). Higher percentages of the horizontal velocity vectors, $P_{H}$, are also present for preterm contraction intervals in the bands B1, B2, and B3. These observations are important and suggest that propagation patterns are quite consistent for dummy intervals in the frequency bands B1, B2, and B3 above 1.0 Hz, where the influence of the maternal heart is expected. For preterm records, in the frequency bands B1, B2, and B3, there is higher electrical activity in the horizontal direction, while for term records, there is higher activity in the vertical direction.

Regarding the ratio between the percentages of velocity vectors in the vertical and horizontal direction, $RP_{\mathrm{VH}}$, it is difficult to conclude anything firm in the frequency bands B0${}^{\prime }$ and Bb. However, in the frequency bands B1, B2, and B3, a really clear pattern is present. The ratios are consistently lower for preterm records, either for dummy or contraction intervals, and are consistently higher for term records, either for dummy or contraction intervals. These results again suggest that the EHG electrical activity in the frequency bands B1, B2, and B3 prevails in the horizontal direction for preterm records, and prevails in the vertical direction for term records.

For the average conduction velocity amplitudes in the vertical, ${\bar{V}}_{V}$, and horizontal, ${\bar{V}}_{H}$, directions, a pattern may be observed. The average conduction velocity amplitudes in the horizontal direction, ${\bar{V}}_{H}$, versus amplitudes in the vertical direction, ${\bar{V}}_{V}$, are higher for preterm and term dummy intervals in the frequency bands B0${}^{\prime }$ (13.4 cm/s vs. 13.3 cm/s and 11.9 cm/s vs. 11.7 cm/s) and Bb (12.7 cm/s vs. 10.8 cm/s and 10.4 cm/s vs. 9.9 cm/s), but are lower for preterm and term contraction intervals in the band B0${}^{\prime }$. In the frequency bands B1, B2, and B3, a clear pattern is again present. The average conduction velocity amplitudes in the vertical direction, ${\bar{V}}_{V}$, versus the amplitudes in the horizontal direction, ${\bar{V}}_{H}$, are higher for both types of intervals and both types of records in the frequency band B1 (9.5 cm/s vs. 9.0 cm/s, 9.2 cm/s vs. 8.9 cm/s, 9.6 cm/s vs. 8.7 cm/s, and 9.3 cm/s vs. 8.9 cm/s), but are lower in the frequency bands B2 and B3 for both types of intervals and both types of records (term contraction intervals in the band B3 are an exception). In terms of conduction velocity amplitudes, dummy intervals exhibit a consistent pattern (higher or lower velocities) for preterm and term records in each frequency band. In addition, the only frequency band where the conduction velocity amplitudes are higher in the vertical direction is the band B1.

Similarly, the ratios between average conduction velocity amplitudes in the vertical and horizontal directions, $RV_{\mathrm{VH}}$, show a clear pattern in the frequency bands B0${}^{\prime }$ and Bb. The ratios are higher in both frequency bands for contraction intervals, either for preterm or term records, than they are for dummy intervals. The velocity ratio, $RV_{\mathrm{VH}}$, is also higher for preterm dummy and contraction intervals in the frequency band B1, and is higher for term dummy and contraction intervals in the frequency band B3.

### 3.3. Percentages and Average Conduction Velocities by Sector

Table 3 summarizes the percentages of velocity vectors and average conduction velocity amplitudes according to individual sectors (south, north, east, and west) in the vertical and horizontal directions for dummy and contraction intervals of preterm and term. Interesting patterns are present in the frequency bands Bb, B1 and B3. In the band Bb, the conduction velocity amplitudes dominate in the south and east sectors. In the band B1, north is the dominating sector (percentages and velocities). In the band B3, the north and west sectors are dominating (percentages and velocities).

### 3.4. Separating Preterm and Term Groups of EHG Records

Table 4 summarizes the significance of each of the newly defined EHG propagation features and of sample entropies of the EHG signals S2, $SE_{S2}$, and S3, $SE_{S3}$, in each of the frequency bands to separate preterm and term dummy and contraction intervals. The *p*-values of the two-sample *t*-test are shown. In addition to sample entropy, $SE_{S2}$(eight times p<0.01), of the newly defined features, the most significant individual features for classification appear to be $P_{H}$ (four times p<0.01), $RP_{\mathrm{VH}}$ (three times p<0.01), ${\bar{V}}_{V}$(five times p<0.01), and ${\bar{V}}_{H}$(three times p<0.01).

**Figure 5:**
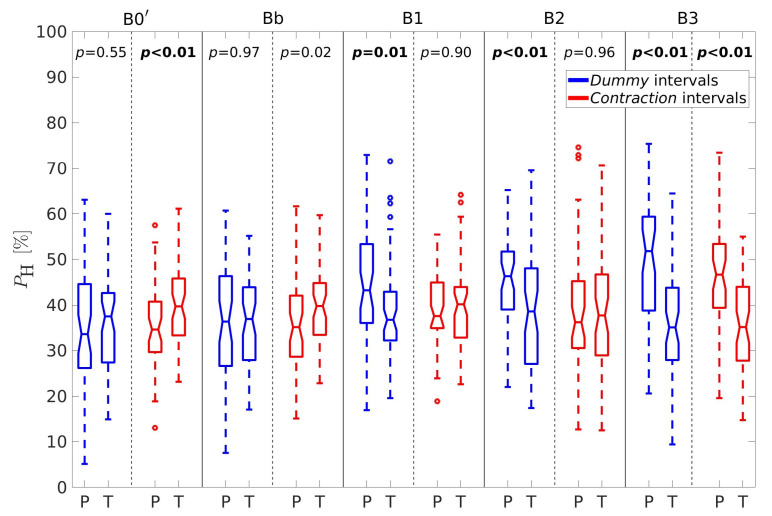
Box plots of the percentages of correlation intervals with velocity vectors in the horizontal direction, $P_{H}$. P—preterm intervals, T—term intervals.

**Figure 6:**
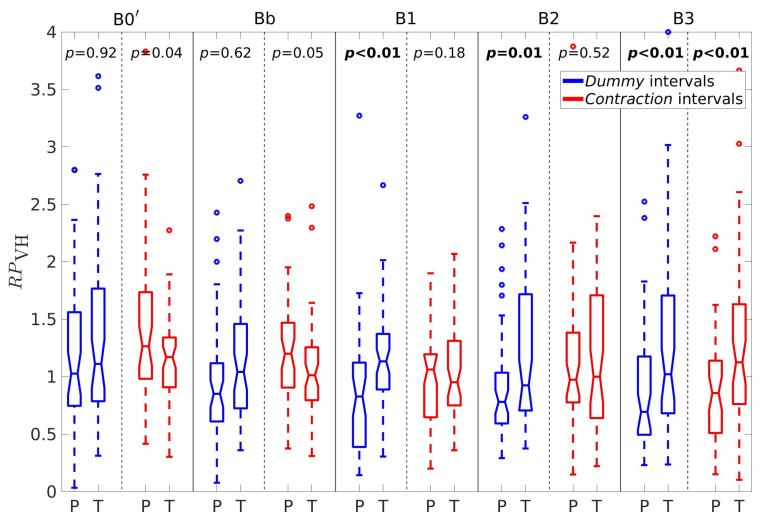
Box plots of the ratio between the percentages of velocity vectors in the vertical and horizontal directions, $RP_{VH}$. P—preterm intervals, T—term intervals.

**Figure 7:**
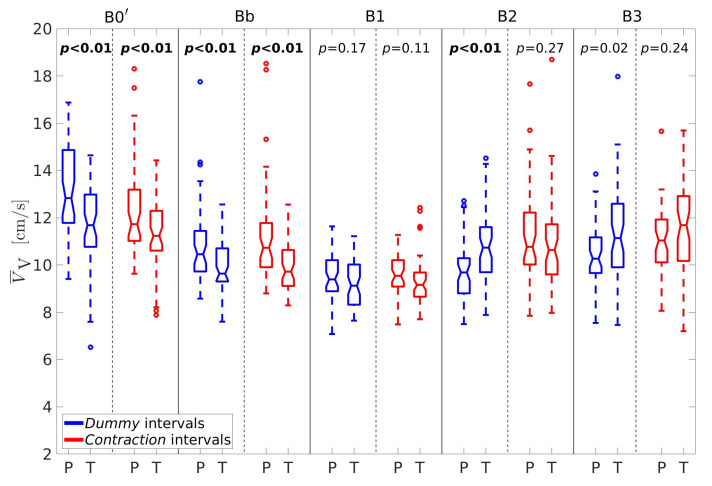
Box plots of the average conduction velocity amplitudes in the vertical direction, ${\bar{V}}_{V}$. P—preterm intervals, T—term intervals.

**Figure 8:**
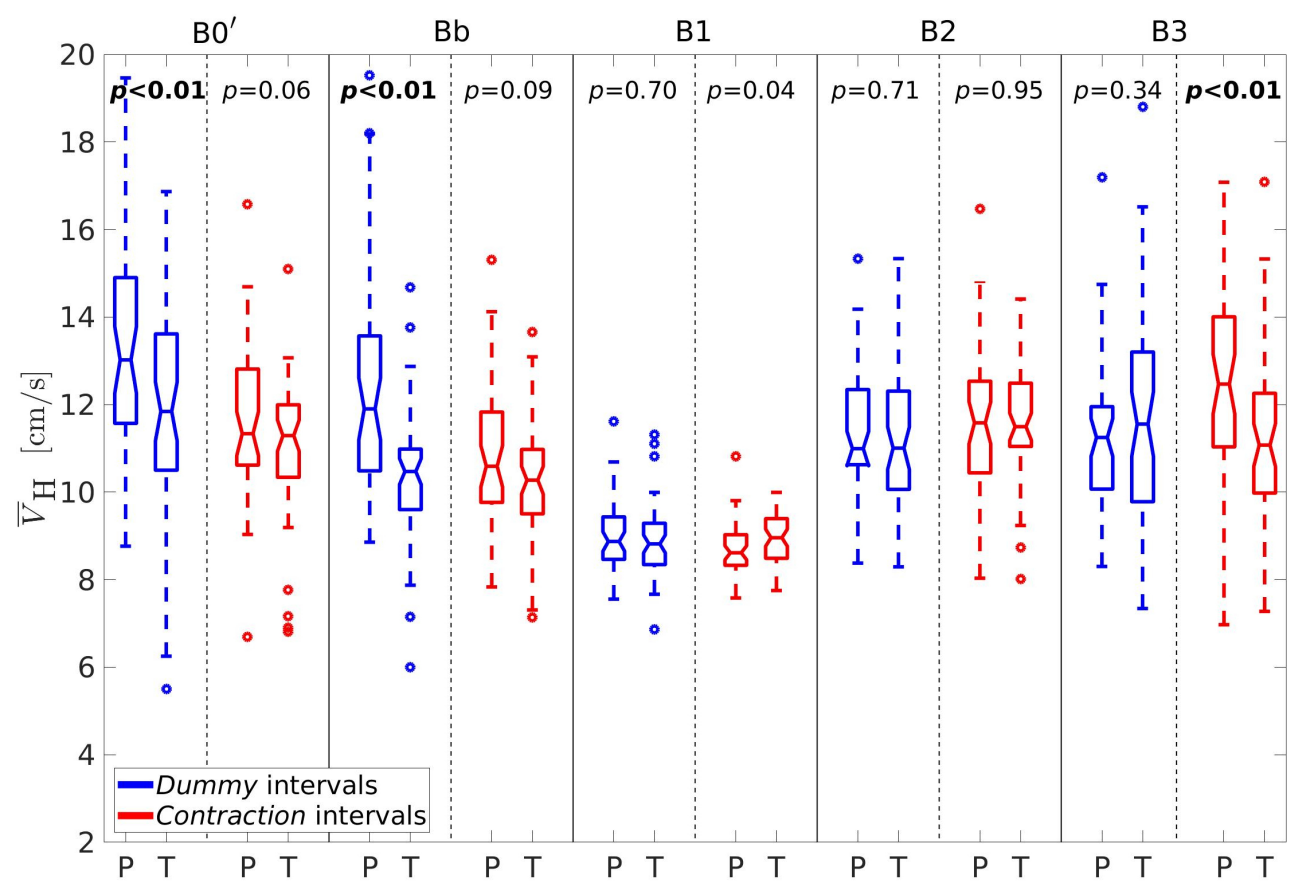
Box plots of the average conduction velocity amplitudes in the horizontal direction, ${\bar{V}}_{H}$. P—preterm intervals, T—term intervals.

**Figure 9:**
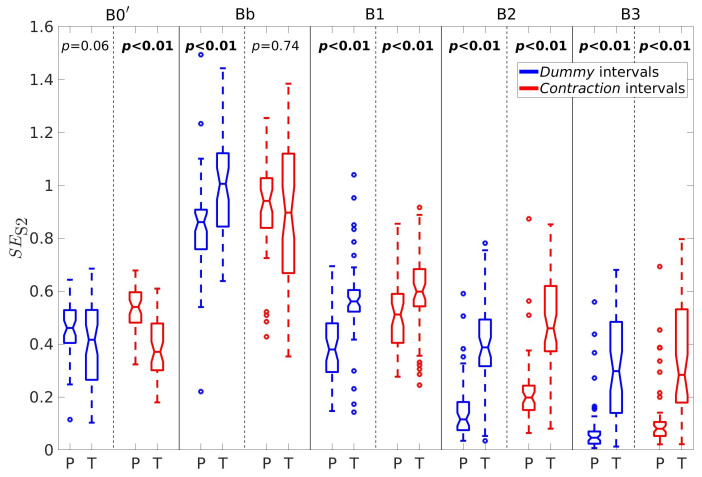
Box plots of sample entropies of the EHG signal S2, $SE_{S2}$. P—preterm intervals, T—term intervals.

For better insight, let us further consider the percentages of horizontal velocity vectors, $P_{H}$, the ratio between the percentages of velocity vectors in the vertical and horizontal direction, $RP_{\mathrm{VH}}$, the average conduction velocity amplitudes in the vertical, ${\bar{V}}_{V}$, and horizontal, ${\bar{V}}_{H}$, directions, and the sample entropy of the EHG signal S2, $SE_{S2}$. Figure 5, Figure 6, Figure 7, Figure 8 and Figure 9 show box plots of $P_{H}$, $RP_{\mathrm{VH}}$, ${\bar{V}}_{V}$, ${\bar{V}}_{H}$, and $SE_{S2}$ for preterm and term groups of dummy and contraction intervals in the frequency bands B0${}^{\prime }$, Bb, B1, B2, and B3 with the corresponding *p*-values. The box plots reveal distributions of feature vectors and their overlap between preterm and term classes.

The percentages of correlation intervals with velocity vectors in the horizontal direction, $P_{H}$(Figure 5), are significantly higher for preterm dummy intervals in comparison to term dummy intervals in the bands B1, B2, and B3. The percentages are also significantly higher for preterm contraction intervals in comparison to term contraction intervals in the band B3, but are significantly lower in the band B0${}^{\prime }$ for these intervals.

There is a similar pattern with the ratios between the percentages of velocity vectors in the vertical and horizontal directions, $RP_{\mathrm{VH}}$(Figure 6). The ratios are significantly lower for preterm dummy intervals in comparison to term dummy intervals in the bands B1, B2, and B3, and are also significantly lower for preterm contraction intervals in comparison to term contraction intervals in the band B3.

The lowest average conduction velocity amplitudes in the vertical direction, ${\bar{V}}_{V}$ (Figure 7), appear to be in the band B1 for both types of intervals and both types of records. The vertical velocities are significantly higher for preterm dummy and contraction, intervals in comparison to term dummy and contraction intervals in the bands B0${}^{\prime }$ and Bb. They are also significantly higher for term dummy intervals versus preterm dummy intervals in the band B2.

There is a similar pattern with the average conduction velocity amplitudes in the horizontal direction, ${\bar{V}}_{H}$(Figure 8). The lowest average conduction velocities appear to be in the band B1 for both types of intervals and both types of records. The horizontal velocities are significantly higher for preterm dummy intervals in comparison to term dummy intervals in the bands B0${}^{\prime }$ and Bb. They are also significantly higher for preterm contraction intervals versus term contraction intervals in the band B3.

The highest sample entropies of the EHG signal S2, $SE_{S2}$ (Figure 9) are in the band Bb. The sample entropies are significantly and consistently lower for preterm dummy and contraction intervals in comparison to term dummy and contraction intervals in the bands B1, B2, and B3. The situation is the same with dummy preterm versus dummy term intervals in the band Bb. However, the sample entropies are significantly higher for preterm contraction intervals versus term contraction intervals in the band B0${}^{\prime }$.

### 3.5. Assessing Individual Classification Accuracies of the Newly Defined Features

Table 5 summarizes the individual classification performances of the newly defined EHG propagation features and of sample entropy features in terms of classifying between preterm and term dummy and contraction intervals. Among the newly defined EHG propagation features, the highest classification accuracy, CA=69.8% and AUC=71.0%, was obtained by the ${\bar{V}}_{H}$ feature in the band Bb for dummy intervals. The highest CA in each frequency band was obtained by the sample entropy of the signal S2, $SE_{S2}$. Considering the classification performance of any individual feature, the CA was higher for contraction intervals (72.6% vs. 61.3%) only in the band B0${}^{\prime }$, while for the rest of the frequency bands, the CA was higher for dummy intervals, i.e., Bb (71.0% vs. 62.3%), B1 (76.4% vs. 60.4%), B2 (84.0% vs. 77.4%), and B3 (78.3% vs. 72.6%). The highest CA of 84.0% was obtained in the band B2 by the $SE_{S2}$ for dummy intervals with Se=84.9%, Sp=83.0%, and AUC=86.4%.

### 3.6. Assessing Classification Accuracies for Combinations of Features

Table 6 summarizes classification accuracies for the selected combinations of features. Neither the combination $P_{V}$ and $P_{H}$ nor the combination ${\bar{V}}_{V}$ and ${\bar{V}}_{H}$ yielded any significant classification performance in classifying between preterm and term dummy and contraction intervals. Higher performances were obtained if combining the newly defined EHG propagation features with the $SE_{S2}$. The following features were taken in combination with the $SE_{S2}$: $RP_{\mathrm{VH}}$, $RV_{\mathrm{VH}}$, $P_{V}$ and $P_{H}$, ${\bar{V}}_{V}$ and ${\bar{V}}_{H}$, and $P_{H}$ and ${\bar{V}}_{V}$. The combination of both sample entropies, $SE_{S2}$ and $SE_{S3}$, was tested for comparison.

In the frequency band B0${}^{\prime }$, the highest CA obtained was 73.6% ($RV_{\mathrm{VH}}$, $SE_{S2}$) for contraction intervals. In the band Bb, the highest CA obtained was 79.3% ($SE_{S2}$, $SE_{S3}$) for dummy intervals. In these two bands, contractions are expected. In the bands B1, B2, and B3, where mainly the influence of the maternal heart on the uterus is expected, the highest CAs were 75.5% ($RV_{\mathrm{VH}}$, $SE_{S2}$) for dummy intervals, 86.8% ($P_{V}$, $P_{H}$, $SE_{S2}$) for dummy intervals, and 84.9% ($RV_{\mathrm{VH}}$, $SE_{S2}$) for dummy intervals, respectively. As for the individual features, the CA obtained by contraction intervals was higher only in the frequency band B0${}^{\prime }$. For the rest of the frequency bands, Bb, B1, B2, and B3, dummy intervals provided higher classification accuracies. Of these, the highest CA obtained was 86.8% ($P_{V}$, $P_{H}$, $SE_{S2}$) for dummy intervals in the band B2 with Se=88.7%, Sp=84.9%, and AUC=90.3%.

## 4. Discussion

Pregnancy is a long process, lasting over nine months. The duration of contraction intervals represents only a small fraction of this period, with other electrical activity and dummy intervals occurring in the remainder. Perhaps an analogy would be useful. Automatic processing of not only abnormal heart beats, but also normal heart beats of an electrocardiogram provides important information for diagnosing many diseases. Similarly, dummy intervals of EHG records provide important information of the state and behavior of the uterus during pregnancy. Thus, characterization and investigation of dummy and contraction intervals and entire EHG records is an important approach for efficient prediction of preterm birth.

The records of the TPEHGT DS contain bipolar EHG signals obtained from four electrodes placed 7 cm apart. We chose this dataset since it contains EHG records recorded early during pregnancy (the mean recording time is the 30th week) and a number of valuable preterm records (the mean delivery time is the 34th week). The unipolar signals are not available with this dataset. Moreover, an advantage of using differential bipolar signals may be recognized in the higher signal quality, allowing more accurate estimation of the uterine voltage peaks. A disadvantage of using unipolar signals may be recognized in the extensive multichannel records required. The interelectrode distance and type of signal (unipolar/bipolar) seem to be important aspects, and will be considered again below.

In the TPEHGT DS, only one term dummy and one term contraction interval relate to a pregnancy (record *tpehgt_p011*) for which the delivery occurred within one week. Assuming the boundary between the non-labor and labor groups of records at one week, all other intervals appear to be in the non-labor phase of pregnancy.

We will first discuss studies that used the maximum likelihood approach for the estimation of the EHG conduction velocities. The first study [35] reported conduction velocities of 7.4 ± 1.5 cm/s and 4.3 ± 0.4 cm/s in the vertical and horizontal directions. The next study, involving 10 women at term with uterine contractions [36], reported conduction velocities of 3.68 ± 3.24 cm/s and 3.76 ± 3.21 cm/s in the vertical and horizontal directions. No obvious or more frequent direction of surface action potential propagation patterns, not even within the same contraction, was found. A preferred direction of propagation of single action potential could not be highlighted. Another study involving 22 pregnant women of which seven were preterm, with a total of 64 contractions [38], reported an average conduction velocity of 8.65 ± 1.90 cm/s for the labor group and of 5.30 ± 1.47 cm/s for the non-labor group. Regarding the direction of propagation, the same conclusion was drawn. The incidence angle of the EHG action potential propagation showed a high variability for both non-labor and labor groups, even within the same contractions. The next study, using 35 contractions of six pregnancies with the elapsed time between the measurement and time of delivery less than or equal to 10 h [41], reported an average propagation velocity of 2.18 ± 0.68 cm/s. The abdominal surface area was also divided into four quadrants. The study reported that 22.9% of contractions originated in the upper part of the uterus, 31.4% in the right part, 20.0% in the lower part, and 25.7% in the left part of the uterus. When dividing the area into only upper and lower parts, 63% of the contractions originated in the upper and 37% in the lower part. Less erratic patterns were found with approaching labor, but no preferred direction of propagation was identified.

A study on EHG propagation analysis during a trial of labor after cesarean section involving 11 pregnancies (six with previous cesarean section and five without) and using the inter-channel correlation between the EHG electric potentials of 64 unipolar electrodes in a grid of 62 × 62 mm [42] reported conduction velocities of 13.73 cm/s for the scar group and 12.14 cm/s for the control group. Concerning the vertical and horizontal propagation directions, variable distributed propagation directions were found as well. Another study involving 89 bursts from eight women who delivered within 18 h [39] estimated conduction velocities and directions by calculating the time difference between the centers of mass of the EHG bursts. This study reported a median velocity of 2.72 (0.95; 8.75) cm/s, and both downward (58%) and upward (42%) propagation directions. No significant differences in the distribution were found, with the conclusion of a multidirectional propagation pattern. The key message of this study was that the labor contractions propagate in both the downward and upward directions in women at term. Yet another study using running cross-correlation windowing on six EHG traces to estimate the time difference between whole contractions, as measured in the upper and lower EHG signals [43], also did not confirm the preferred dominant downward propagation direction. A certain number of contractions with opposite propagation direction occurred, and the estimated conduction velocity was 2.3 cm/s.

In a study that was based on the visual estimation of conduction velocities [10], the propagation velocity of the EHG signals was estimated as the time difference between two bipolar signals arriving from two electrode pairs (the upper and lower EHG signals). Only conduction velocities in the vertical direction were estimated. Upward and downward directions were not differentiated. The estimated conduction velocities were 11.11 ± 5.13 cm/s and 11.31 ± 2.89 cm/s for preterm and term non-labor groups, and were 52.56 ± 33.94 cm/s and 31.25 ± 14.91 cm/s for preterm and term labor groups of pregnant women. The boundary between the non-labor and labor groups was set at seven days.

Studies on evaluating the synchronization and directionality of uterine EMG signals [34] and on estimating the coupling and directionality of the EHG signals [44] reported no dominant direction in propagation patterns of signals for contractions present four weeks before labor or for three weeks before labor, but with a dominant direction towards the cervix for labor contractions in both studies.

All of these findings seem to be in accordance with our findings. The aggregate average conduction velocities in the, e.g., frequency band Bb (Table 1), were 8.93 ± 0.41 cm/s and 8.78 ± 0.37 cm/s for preterm and term dummy intervals, and were 9.03 ± 0.49 cm/s and 8.86 ± 0.28 cm/s for preterm and term contraction intervals. The high values of the estimated conduction velocities in [10] may be due to the use of only two (bipolar) signals, which only offered estimation in the vertical direction [37,40]. The higher values of the estimated conduction velocities in our study, using the short-time cross-correlation technique, as compared to the velocities estimated in the studies using a grid of unipolar signals and the maximum likelihood approach [35,36,38,41], may be due to our use of only four (bipolar) signals measured from four electrodes 7 cm apart, the assumption of linear propagation of the EHG waves for each correlation interval, and different conduction-velocity estimation technique. On the other hand, in the study using inter-channel correlation between the EHG electric potentials of 64 unipolar electrodes in the grid of 62 × 62 mm [42], higher conduction velocities in comparison to ours were reported. At this point, inter-electrode distances and the type of signal (unipolar/bipolar) need to be considered. According to the comparisons discussed, it does not seem that the inter-electrode distances or the type of signal has a high impact on the estimation. In addition, the optimal inter-electrodes distances have not been established [40], nor has the impact of the type of signal on the estimation been investigated in detail. In any case, our main goal was not to estimate the precise conduction velocities. Our main goal was a robust estimation of the percentages and average conduction velocities in the vertical and horizontal directions, together with their ratios, in different frequency bands, and the identification of useful new features for separating between preterm and term EHG records.

We did not find any major or preferred continuous direction of propagation of the EHG waves according to directions or sectors in any of the frequency bands for either dummy or contraction intervals. The propagation directions were highly variable and distributed. Moreover, no obvious pattern or trajectory of velocity vectors was found. These results are in accordance with the findings of other studies [36,38,39,41,42,43]. The only two studies that reported the dominant direction towards the cervix [34,44] estimated it for labor contractions. Furthermore, small differences in the average conduction velocity amplitudes in the vertical and horizontal directions (Table 2 and Table A2) do occur. Finally, in our study, on average, a significantly higher percentage of velocity vectors in the horizontal direction and a significantly lower percentage of velocity vectors in the vertical direction were found for preterm dummy intervals (Table 2) in the frequency bands B1, B2, and B3 above 1.0 Hz. This is an important new marker. We will try to explain why this change in percentages happens in the last paragraph of the Conclusion section.

The TPEHGT DS contains 47 human-annotated preterm dummy and contraction intervals and 53 human-annotated term dummy and contraction intervals. In order to balance both classes, the SMOTE technique was used, increasing the number of examples in the minority preterm class from 47 to 53. Over-sampling did not seem to have had a high impact on the performance, since the numbers of examples in both classes are pretty close. The highest classification performances obtained by newly defined individual features (Table 5) were CA=69.8% and AUC=72.0% when using the average velocity in the vertical direction, ${\bar{V}}_{H}$, in the frequency band Bb for dummy intervals. Among all individual features, the sample entropy, $SE_{S2}$, estimating the entropy of the EHG activity in the vertical direction, yielded the highest performance of CA=84.0% and AUC=86.4% in the band B2, again for dummy intervals. However, when using pairs or simple combinations of features (Table 6), the highest performances obtained were Se=88.7%, Sp=84.9%, CA=86.8%, and AUC=90.3% in the band B2 for dummy intervals using the $P_{V}$, $P_{H}$, and $SE_{S2}$ features. The percentages of velocity vectors in the vertical, $P_{V}$, and horizontal, $P_{H}$, directions in the frequency region above 1.0 Hz proved to be significant and valuable markers for differentiating between preterm and term EHG records, and help to further understand the behavior of the uterus during pregnancy. The following combinations of features also appear to be powerful: $RV_{\mathrm{VH}}$ and $SE_{S2}$ (band B3, dummy intervals, CA=84.9%, AUC=89.6%), $P_{H}$, ${\bar{V}}_{V}$, and $SE_{S2}$ (band B3, dummy intervals, CA=84.9%, AUC=90.2%), or $SE_{S2}$ and $SE_{S3}$ (band B2, dummy intervals, CA=85.8%, AUC=89.1%). Moreover, dummy intervals in the bands Bb, B1, B2, and B3 resulted in higher classification performances than contraction intervals. These results confirm that dummy intervals and the frequency region above 1.0 Hz, where the maternal heart influence on the uterus is present, are equally (or even more) important for predicting preterm birth as contraction intervals are. If using this “dummy” approach, there is no need to wait for or to seek contraction intervals during clinical investigation. Clinical investigation can also be performed early during pregnancy, e.g., around the 30th week of pregnancy (the mean recording time of the EHG records of the TPEHGT DS) when contraction intervals may or may not be present.

It would be possible to further employ one of the machine-learning feature-selection techniques in order to seek an optimal subset of newly defined features. Our preliminary attempt resulted in 12 selected features from dummy intervals and from the frequency bands above 1.0 Hz, with a CA of 93.4% and AUC of 96.3%. However, these feature selection techniques may be unstable, resulting in different subsets of the selected features after each trial. In any case, the goal of this study was not to achieve as high a classification accuracy as possible at the cost of stability or over-estimation of the actual performance. The goal was to find simple new features that could be considered as new biophysical markers to predict preterm birth, with as accurate, and still acceptable, classification performance as possible.

Next, we try to compare the obtained classification performances of individual features with the performances obtained by other studies that also investigated individual features for the classification between preterm and term deliveries. A study investigating individual classification accuracies of sample entropy using a database of 120 women admitted to a hospital for preterm contractions, four unipolar signals, modified sample entropy, approximate entropy, modified approximate entropy, and time reversibility in terms of preterm vs. term contraction segments, as well as five-fold cross-validation [6], reported a maximum classification accuracy of 75% for modified approximate entropy. The next study, which used the TPEHG DB and three bipolar signals to investigate classification accuracies of single-wavelet-based features in terms of single preterm vs. term subsegments (6.8 min) of entire EHG records through leave-one-out cross-validation [22], reported a maximum classification accuracy of 71%. It is difficult to compare these results or to determine the best single feature, since the researchers of each of these studies used their own datasets, and the number of examples per dataset differed.

In a previous study [5], we attempted to describe the electro-mechanical activity of a pregnant uterus within dummy intervals out of contractions due to the influence of the maternal heart. The present study confirmed that the propagation velocities of preterm and term dummy and contraction intervals and of entire records are approximately equal (Table 1, Table A1 and Table A2), and can be excluded as a property causing changes in the influence of the maternal heart on the uterus. In the previous study [5], we hypothesized the following: (1) During the term non-labor phase of pregnancy, while the cervix is unripe and rigid, the electrical pulses caused by the maternal heart activity in the frequency band B1 (maternal heart rate) propagate in the vertical direction along the uterine muscle, and are reflected back due to the discontinuity (closed womb), causing interference with themselves, and strong higher harmonics in the bands B2 and B3 appear; and (2) during the term labor phase, and during preterm non-labor and labor phases of pregnancy, while the cervix effaces and slowly dilates, the electrical pulses diffract through the hole opening, and their intensity in the vertical direction diminishes or remains low throughout, together with higher harmonics. Our present findings seem to be in agreement with this model. For term dummy intervals, during the interference phase, there is an approximately equal, and slightly higher, percentage of velocity vectors in the vertical direction, $P_{V}$, in comparison to the horizontal direction, $P_{H}$, in the frequency bands B1, B2, and B3 (see Table 2). Since the signal S2 is “reached” with the higher harmonics, it is less regular, resulting in high sample entropies in the frequency bands B1, B2, and B3 (see Figure 9). For preterm dummy intervals, during the diffraction phase, there is a significantly lower percentage of velocity vectors in the vertical direction, $P_{V}$, and significantly higher in the horizontal direction, $P_{H}$, in the frequency bands B1, B2, and B3 (Table 2). Since the signal S2 appears to be “poor” with the higher harmonics, it is more regular, resulting in low sample entropies in the frequency bands B1, B2, and B3 (Figure 9). The sample entropies, $SE_{S2}$, are also much lower for preterm dummy intervals than they are for term dummy intervals in each of the frequency bands B1, B2, and B3 (Figure 9).

## 5. Conclusions

The main purposes of this study were assessing the velocity and directionality of uterine electrical activity using EHG surface records and the establishment and validation of new features for efficient preterm birth prediction. The innovations brought are:

1. Development of a relatively simple short-time cross-correlation technique using bipolar EHG surface signals for estimating and characterizing the conduction velocity amplitudes and directions of the EHG surface electric potential propagation;
2. Confirmation of the hypothesis that the newly defined features—percentages of the conduction velocities in the vertical, $P_{V}$, and horizontal, $P_{H}$, directions extracted from the frequency bands B1, B2, and B3 and above 1.0 Hz, where the electrical influence of the maternal heart on the uterus is present—are very useful features or markers for helping predicting preterm birth;
3. Confirmation of the hypothesis [5] that dummy intervals of the EHG records are equally as, or even more, important for predicting preterm birth as contraction intervals are;
4. Justification that the percentages of conduction velocity vectors in the vertical, $P_{V}$, and horizontal, $P_{H}$, directions in combination with the sample entropy of the EHG signal S2, $SE_{S2}$, recorded in the vertical direction and obtained from dummy intervals above 1.0 Hz are powerful features for distinguishing between preterm and term EHG surface records.

We hope that the findings described in this study will result in new studies exploring the further understanding of the physiological mechanisms of the uterus that are involved during pregnancy. Further investigations using machine-learning feature-selection techniques in order to seek an optimal set of features and higher classification accuracies are also possible.

## Figures and Tables

**Table 1:** Aggregate average conduction velocity amplitudes, $\bar{CV}$, for dummy and contraction intervals, and for entire EHG records. The highest velocity amplitudes per frequency band, per type of interval, and per type of records are shaded in gray.

				$\bar{\mathrm{CV}}$ [cm/s]		
		B0${}^{\prime }$	Bb	B1	B2	B3
**Dummy intervals**	Preterm	9.25 ± 0.58	8.93 ± 0.41	8.42 ± 0.55	9.37 ± 0.42	9.45 ± 0.53
	Term	9.04 ± 0.31	8.78 ± 0.37	8.45 ± 0.73	9.42 ± 0.33	9.55 ± 0.41
**Contraction intervals**	Preterm	9.47 ± 0.44	9.03 ± 0.49	8.03 ± 0.75	9.32 ± 1.07	9.39 ± 1.08
	Term	9.19 ± 0.50	8.86 ± 0.28	8.31 ± 0.60	9.48 ± 0.47	9.67 ± 0.52
**Entire records**	Preterm	9.36 ± 0.40	8.96 ± 0.27	8.29 ± 0.54	9.29 ± 0.68	9.51 ± 0.60
	Term	9.08 ± 0.27	8.83 ± 0.13	8.44 ± 0.47	9.40 ± 0.29	9.63 ± 0.44

**Table 2:** Percentages of correlation intervals with the velocity vector in the vertical, $P_{V}$, and horizontal, $P_{H}$, directions, their ratios, $RP_{VH}$, the average conduction velocity amplitudes in the vertical, ${\bar{V}}_{V}$, and horizontal, ${\bar{V}}_{H}$, directions, and their ratios, $RV_{VH}$. All statistics are aggregate averages over all dummy and contraction intervals. The highest percentages (and the highest velocities) per vertical or horizontal direction, per type of interval, and per type of record are shaded in gray. The highest two ratios of the percentages (and of velocities) per frequency band are shaded in gray and in bold.

Band	Intervals	Records	$P_{V}$	$P_{H}$	${\mathrm{RP}}_{\mathrm{VH}}$	${\bar{V}}_{V}$	${\bar{V}}_{H}$	${\mathrm{RV}}_{\mathrm{VH}}$
			[%]	[%]		[cm/s]	[cm/s]	
**B0${}^{\prime }$**	Dummy	Preterm	34.9 ± 14	34.5 ± 14	**1.40 ± 1.12**	13.3 ± 2.1	13.4 ± 2.6	1.01 ± 0.17
		Term	41.7 ± 12	36.0 ± 11	**1.38 ± 0.78**	11.7 ± 1.8	11.9 ± 2.4	1.01 ± 0.18
	Contraction	Preterm	43.0 ± 10	35.1 ± 9.2	1.36 ± 0.63	12.5 ± 2.4	11.7 ± 1.7	**1.08 ± 0.20**
		Term	43.4 ± 7.6	40.1 ± 7.9	1.15 ± 0.37	11.3 ± 1.5	11.1 ± 1.6	**1.03 ± 0.13**
**Bb**	Dummy	Preterm	29.7 ± 12	35.9 ± 13	1.05 ± 1.07	10.8 ± 1.7	12.7 ± 3.0	0.88 ± 0.17
		Term	36.7 ± 10	36.0 ± 9.8	**1.14 ± 0.54**	9.9 ± 1.1	10.4 ± 1.5	0.96 ± 0.14
	Contraction	Preterm	39.4 ± 8.6	35.0 ± 9.6	**1.24 ± 0.48**	11.3 ± 2.0	10.8 ± 1.6	**1.05 ± 0.18**
		Term	38.8 ± 8.5	39.2 ± 8.2	1.06 ± 0.41	9.9 ± 1.0	10.3 ± 1.4	**0.97 ± 0.15**
**B1**	Dummy	Preterm	32.7 ± 13	45.2 ± 13	0.86 ± 0.56	9.5 ± 0.9	9.0 ± 0.8	**1.07 ± 0.14**
		Term	40.8 ± 7.1	38.5 ± 10	**1.15 ± 0.40**	9.2 ± 1.0	8.9 ± 0.8	1.04 ± 0.13
	Contraction	Preterm	34.5 ± 10	39.1 ± 7.9	0.95 ± 0.39	9.6 ± 0.8	8.7 ± 0.6	**1.11 ± 0.11**
		Term	38.5 ± 7.7	39.3 ± 9.5	**1.06 ± 0.39**	9.3 ± 1.0	8.9 ± 0.6	1.04 ± 0.12
**B2**	Dummy	Preterm	36.5 ± 10	45.4 ± 9.7	0.90 ± 0.47	9.7 ± 1.3	11.3 ± 1.4	0.87 ± 0.16
		Term	40.6 ± 13	38.2 ± 12	**1.22 ± 0.66**	10.9 ± 2.1	11.2 ± 1.6	**1.00 ± 0.22**
	Contraction	Preterm	36.5 ± 10	39.0 ± 13	1.11 ± 0.63	11.3 ± 1.9	11.6 ± 1.7	**0.98 ± 0.16**
		Term	38.2 ± 13	38.9 ± 13	**1.21 ± 0.91**	10.9 ± 1.8	11.6 ± 1.3	0.94 ± 0.19
**B3**	Dummy	Preterm	37.2 ± 12	49.4 ± 13	0.88 ± 0.53	10.5 ± 1.3	11.3 ± 1.7	0.94 ± 0.17
		Term	39.1 ± 14	36.0 ± 12	**1.28 ± 0.79**	11.3 ± 2.0	11.7 ± 2.4	**1.01 ± 0.27**
	Contraction	Preterm	35.3 ± 11	46.6 ± 13	0.86 ± 0.44	11.1 ± 1.4	12.5 ± 2.2	0.92 ± 0.24
		Term	38.1 ± 13	35.4 ± 10	**1.25 ± 0.76**	11.5 ± 1.9	11.2 ± 1.8	**1.05 ± 0.22**

**Table 3:** The percentages of velocity vectors, $P_{S}$ (south), $P_{N}$ (north), $P_{E}$ (east), and $P_{W}$ (west), and average conduction velocity amplitudes, ${\bar{V}}_{S}$ (south), ${\bar{V}}_{N}$ (north), ${\bar{V}}_{E}$ (east), and ${\bar{V}}_{W}$ (west), by sector in the vertical and horizontal directions. All statistics are aggregate average statistics over all dummy and contraction intervals. The highest percentage per vertical (or per horizontal) direction and the highest average conduction velocity per vertical (or horizontal) direction is shaded in gray.

			Vertical		Horizontal		Vertical		Horizontal	
Band	Intervals	Records	$P_{S}$	$P_{N}$	$P_{E}$	$P_{W}$	${\bar{V}}_{S}$	${\bar{V}}_{N}$	${\bar{V}}_{E}$	${\bar{V}}_{W}$
			[%]	[%]	[%]	[%]	[cm/s]	[cm/s]	[cm/s]	[cm/s]
**B0${}^{\prime }$**	Dummy	Preterm	17.2	17.7	18.1	16.4	12.74	13.04	13.36	13.35
		Term	21.2	20.5	18.1	17.9	11.64	11.79	12.00	11.79
	Contraction	Preterm	20.3	22.7	17.0	18.1	12.30	12.21	11.57	11.66
		Term	20.8	22.5	19.8	20.2	11.21	11.38	10.92	11.19
**Bb**	Dummy	Preterm	14.9	14.8	18.6	17.4	10.93	10.34	12.69	12.61
		Term	18.1	18.6	18.3	17.8	10.19	9.50	10.44	10.41
	Contraction	Preterm	19.6	19.9	17.2	17.8	11.46	11.06	10.91	10.62
		Term	19.3	19.4	19.2	20.1	10.01	9.72	10.29	10.28
**B1**	Dummy	Preterm	16.2	16.4	22.0	23.3	9.49	9.48	9.28	8.72
		Term	19.9	20.9	20.2	18.3	9.17	9.26	8.96	9.11
	Contraction	Preterm	17.0	17.5	20.2	18.9	9.59	9.48	8.74	8.65
		Term	18.5	19.9	20.9	18.4	9.28	9.25	8.64	9.44
**B2**	Dummy	Preterm	19.0	17.5	23.6	21.8	9.87	9.58	11.28	11.33
		Term	18.9	21.6	18.7	19.6	10.82	10.90	11.60	10.66
	Contraction	Preterm	18.8	17.7	20.4	18.6	10.99	11.37	11.60	11.54
		Term	17.5	20.7	19.6	19.3	10.77	10.79	11.95	11.34
**B3**	Dummy	Preterm	18.2	19.0	22.2	27.2	9.93	10.90	9.70	12.54
		Term	19.3	19.8	16.0	20.0	10.76	11.73	10.71	12.38
	Contraction	Preterm	17.3	18.0	19.5	27.2	9.78	11.92	10.25	14.16
		Term	18.0	20.0	15.0	20.4	10.69	12.04	10.34	11.80

**Table 4:** *p*-values of newly defined EHG propagation features and of sample entropy of the original EHG signals S2, $SE_{S2}$, and S3, $SE_{S3}$. *p*-values ≤0.01 are shaded in gray.

					*p*-Values				
Band	Intervals	$P_{V}$	$P_{H}$	${\mathrm{RP}}_{\mathrm{VH}}$	${\bar{V}}_{V}$	${\bar{V}}_{H}$	${\mathrm{RV}}_{\mathrm{VH}}$	${\mathrm{SE}}_{S2}$	${\mathrm{SE}}_{S3}$
**B0${}^{\prime }$**	Dummy	**0.01**	0.55	0.92	**<0.01**	**<0.01**	0.89	0.06	0.35
	Contraction	0.82	**<0.01**	0.04	**<0.01**	0.06	0.19	**<0.01**	**<0.01**
**Bb**	Dummy	**<0.01**	0.97	0.62	**<0.01**	**<0.01**	**0.01**	**<0.01**	0.62
	Contraction	0.71	0.02	0.05	**<0.01**	0.09	0.02	0.74	0.03
**B1**	Dummy	**<0.01**	**0.01**	**<0.01**	0.17	0.70	0.39	**<0.01**	0.35
	Contraction	0.03	0.90	0.18	0.11	0.04	**0.01**	**<0.01**	0.14
**B2**	Dummy	0.09	**<0.01**	**0.01**	**<0.01**	0.71	**<0.01**	**<0.01**	0.58
	Contraction	0.48	0.96	0.52	0.27	0.95	0.33	**<0.01**	0.02
**B3**	Dummy	0.47	**<0.01**	**<0.01**	0.02	0.34	0.17	**<0.01**	0.12
	Contraction	0.27	**<0.01**	**<0.01**	0.24	**<0.01**	**0.01**	**<0.01**	0.13

**Table 5:** Classification performance results obtained using individual features for preterm and term dummy and contraction intervals of the TPEHGT DS. The highest three classification accuracies, CA, per dummy or contraction interval are shaded in gray. The highest CA per frequency band is shaded in gray and in bold. The highest CA is shaded in dark grey.

Band	Intervals	Measure [%]	$P_{V}$	$P_{H}$	${\mathrm{RP}}_{\mathrm{VH}}$	${\bar{V}}_{V}$	${\bar{V}}_{H}$	${\mathrm{RV}}_{\mathrm{VH}}$	${\mathrm{SE}}_{S2}$	${\mathrm{SE}}_{S3}$
**B0${}^{\prime }$**	Dummy	*Se*	35.9	39.6	20.8	50.9	52.8	45.3	79.3	79.3
		*Sp*	69.8	67.9	86.8	67.9	69.8	44.4	41.5	37.7
		*CA*	52.8	53.8	53.8	59.4	61.3	44.9	60.4	58.5
		*AUC*	52.4	56.0	50.4	67.3	65.9	42.9	64.7	55.2
**B0${}^{\prime }$**	Contraction	*Se*	41.5	41.5	37.7	28.3	49.1	22.2	75.5	79.3
		*Sp*	73.6	69.8	83.0	73.6	52.8	79.3	69.8	56.6
		*CA*	57.6	55.7	60.4	50.9	50.9	50.5	**72.6**	67.9
		*AUC*	59.4	61.0	61.0	48.0	53.6	49.7	76.8	68.9
**Bb**	Dummy	*Se*	39.6	39.6	24.5	45.3	52.8	52.8	75.9	86.8
		*Sp*	73.6	71.7	92.5	79.3	86.8	71.7	66.0	54.7
		*CA*	56.6	55.7	58.5	62.3	69.8	62.3	**71.0**	70.8
		*AUC*	54.9	59.8	53.4	65.7	72.0	62.6	73.1	72.2
**Bb**	Contraction	*Se*	32.1	39.6	28.3	37.7	52.8	30.2	71.7	75.5
		*Sp*	49.1	71.7	79.3	86.8	62.3	66.0	39.6	43.4
		*CA*	40.6	55.7	53.8	62.3	57.6	48.1	55.7	59.4
		*AUC*	35.9	58.1	54.2	59.3	58.0	45.6	54.9	59.6
**B1**	Dummy	*Se*	41.5	30.2	39.6	28.3	54.7	35.9	69.8	81.1
		*Sp*	84.9	84.9	77.4	50.9	45.3	62.3	83.0	46.3
		*CA*	63.2	57.6	58.5	39.6	50.0	49.1	**76.4**	63.6
		*AUC*	58.8	58.3	58.4	35.8	47.6	46.9	75.4	71.0
**B1**	Contraction	*Se*	45.3	58.5	52.8	64.2	39.6	41.5	67.9	77.4
		*Sp*	73.6	35.9	45.3	37.7	67.9	71.7	52.8	41.5
		*CA*	59.4	47.2	49.1	50.9	53.8	56.6	60.4	59.4
		*AUC*	57.1	43.9	57.4	46.7	53.9	57.7	65.6	63.5
**B2**	Dummy	*Se*	77.4	77.4	84.9	81.1	62.3	77.4	84.9	77.4
		*Sp*	39.6	49.1	37.7	49.1	41.5	50.9	83.0	49.1
		*CA*	58.5	63.2	61.3	65.1	51.9	65.1	**84.0**	63.2
		*AUC*	53.3	62.8	55.2	68.0	48.6	67.6	86.4	73.6
**B2**	Contraction	*Se*	73.6	34.0	83.0	24.5	28.3	62.3	81.1	81.1
		*Sp*	43.4	56.6	22.6	69.8	71.7	32.1	73.6	39.6
		*CA*	58.5	45.3	52.8	47.2	50.0	47.2	77.4	60.4
		*AUC*	60.5	41.8	53.9	42.3	47.7	42.2	83.8	56.8
**B3**	Dummy	*Se*	69.8	60.4	81.5	79.3	86.8	83.0	92.5	77.4
		*Sp*	39.6	69.8	37.7	43.4	39.6	47.2	64.2	39.6
		*CA*	54.7	65.1	59.8	61.3	63.2	66.0	**78.3**	58.5
		*AUC*	54.0	70.4	54.5	62.3	60.3	71.1	84.5	60.2
**B3**	Contraction	*Se*	67.9	60.4	88.7	75.5	54.7	67.9	88.7	73.6
		*Sp*	49.1	73.6	37.7	39.6	73.6	62.3	56.6	32.1
		*CA*	58.5	67.0	63.2	57.6	64.2	65.1	72.6	52.8
		*AUC*	57.7	76.4	66.0	58.5	67.1	68.9	81.6	49.5

**Table 6:** Classification performance results obtained using combinations of features for preterm and term dummy and contraction, intervals of the TPEHGT DS. The highest three classification accuracies, CA, per dummy or contraction interval are shaded in gray. The highest CA per frequency band is shaded in gray and in bold. The highest CA is shaded in dark grey.

Band	Intervals	Measure [%]	$P_{V}$,$P_{H}$	${\bar{V}}_{V}$,${\bar{V}}_{H}$	${\mathrm{RP}}_{\mathrm{VH}}$	${\mathrm{RV}}_{\mathrm{VH}}$	$P_{V}$,$P_{H}$	${\bar{V}}_{V}$,${\bar{V}}_{H}$	$P_{H}$,${\bar{V}}_{V}$	
					${\mathrm{SE}}_{S2}$	${\mathrm{SE}}_{S2}$	${\mathrm{SE}}_{S2}$	${\mathrm{SE}}_{S2}$	${\mathrm{SE}}_{S2}$	${\mathrm{SE}}_{S2}$,${\mathrm{SE}}_{S3}$
**B0${}^{\prime }$**	Dummy	*Se*	47.2	58.5	37.7	77.4	67.9	64.2	71.7	81.1
		*Sp*	69.8	66.0	77.4	43.4	69.8	75.5	71.7	41.5
		*CA*	58.5	62.3	57.6	60.4	68.9	69.8	71.7	61.3
		*AUC*	62.5	67.5	69.0	62.0	77.2	75.7	77.7	59.6
**B0${}^{\prime }$**	Contraction	*Se*	39.6	30.2	77.4	77.4	73.6	69.8	73.6	75.5
		*Sp*	77.4	75.5	66.0	69.8	71.7	69.8	71.7	67.9
		*CA*	58.5	52.8	71.7	**73.6**	72.6	69.8	72.6	71.7
		*AUC*	60.2	53.8	77.4	76.6	81.8	73.6	78.3	76.4
**Bb**	Dummy	*Se*	47.2	50.9	28.3	62.3	69.8	60.4	62.3	84.9
		*Sp*	67.9	86.8	90.6	75.5	61.1	86.8	77.4	73.6
		*CA*	57.6	68.9	59.4	68.9	65.4	73.6	69.8	**79.3**
		*AUC*	62.7	73.8	71.4	75.7	76.6	80.0	78.0	84.3
**Bb**	Contraction	*Se*	35.9	34.0	54.7	54.7	50.9	50.9	52.8	67.9
		*Sp*	69.8	84.9	62.3	67.9	69.8	81.1	81.1	71.7
		*CA*	52.8	59.4	58.5	61.3	60.4	66.0	67.0	69.8
		*AUC*	53.7	60.9	63.8	66.9	70.0	70.0	68.2	73.7
**B1**	Dummy	*Se*	41.5	62.3	69.8	71.7	66.0	71.7	66.0	75.5
		*Sp*	83.0	41.5	77.4	79.3	81.1	73.6	71.7	75.5
		*CA*	62.3	51.9	73.6	**75.5**	73.6	72.6	68.9	75.4
		*AUC*	56.6	50.9	75.0	76.6	79.5	75.7	72.1	82.7
**B1**	Contraction	*Se*	37.7	47.2	73.6	71.7	66.0	71.7	71.7	64.2
		*Sp*	67.9	58.5	52.8	64.2	56.6	52.8	60.4	64.2
		*CA*	52.8	52.8	63.2	67.9	61.3	62.3	66.0	64.2
		*AUC*	53.7	53.6	67.7	73.3	65.0	71.5	72.2	68.0
**B2**	Dummy	*Se*	79.3	77.4	83.0	88.7	88.7	88.7	84.9	88.7
		*Sp*	66.0	47.2	77.4	77.4	84.9	75.5	77.4	83.0
		*CA*	72.6	62.3	80.2	83.0	**86.8**	82.1	81.1	85.8
		*AUC*	77.6	65.7	85.9	88.2	90.3	87.4	88.1	89.1
**B2**	Contraction	*Se*	66.0	30.2	77.4	81.1	73.6	81.1	81.1	84.9
		*Sp*	49.1	73.6	66.0	73.6	75.5	79.3	67.9	81.1
		*CA*	57.6	51.9	71.7	77.4	74.5	80.2	74.5	83.0
		*AUC*	58.4	52.0	82.0	86.9	85.2	85.7	82.7	87.6
**B3**	Dummy	*Se*	60.4	79.3	88.7	88.7	84.9	86.8	90.6	92.5
		*Sp*	62.3	54.7	66.0	81.1	67.9	75.5	79.3	64.2
		*CA*	61.3	67.0	77.4	**84.9**	76.4	81.1	**84.9**	78.3
		*AUC*	69.4	72.3	85.5	89.6	85.1	89.5	90.2	82.5
**B3**	Contraction	*Se*	66.0	62.3	90.6	84.9	84.9	84.9	84.9	88.7
		*Sp*	71.7	71.7	60.4	66.0	67.9	66.0	71.7	56.6
		*CA*	68.9	67.0	75.5	75.5	76.4	75.5	78.3	72.6
		*AUC*	76.5	71.6	81.7	85.5	84.7	85.4	85.9	81.5

## Appendix A

The measure to estimate the discrepancies between the actually measured and calculated signal S4 was the standard deviation between the samples of signals,

(A1) $$\sigma^{\;2}\;\;=\;\;\frac{1}{N}\;\sum_{n=0}^{N-1}\;\;{\left(\left(S4_{M}\left(n\right)-\mu_{M}\right)-\left(S4_{C}\left(n\right)-\mu_{C}\right)\right)}^{2}\;,$$

where $S4_{M}$ and $S4_{C}$ are the measured and calculated signal S4, $\mu_{M}$ and $\mu_{C}$ are the means of measured and calculated signals, and *N* is the number of signal samples. An example of an excerpt of the measured and calculated signal S4 is shown in Figure A1. The discrepancies between measured and calculated signal S4 were estimated for a few preterm and term EHG records. Standard deviations according to Equation (A1) were: 0.82 (μV)${}^{2}$, 0.79 (μV)${}^{2}$, and 1.06 (μV)${}^{2}$ for preterm records, and were: 0.87 (μV)${}^{2}$, 1.05 (μV)${}^{2}$, and 0.78 (μV)${}^{2}$ for term records, and are negligible.

## Appendix C

**Table A1:** Average conduction velocity amplitudes, $\bar{CV}$, as estimated from preterm and term dummy and contraction intervals of the EHG records of the TPEHGT DS.

				$\bar{\mathrm{CV}}$ [cm/s]		
Preterm Records	Intervals	B0${}^{\prime }$	Bb	B1	B2	B3
*tpehgt_p001*	Dummy	8.7 ± 4.8	8.5 ± 4.6	9.1 ± 4.5	9.7 ± 5.3	9.5 ± 6.0
	Contraction	9.3 ± 5.1	8.7 ± 4.6	9.0 ± 4.5	9.3 ± 5.7	9.1 ± 5.9
*tpehgt_p002*	Dummy	9.8 ± 6.6	9.1 ± 6.1	7.8 ± 4.6	9.4 ± 5.8	9.8 ± 6.4
	Contraction	9.2 ± 6.4	8.7 ± 5.8	7.7 ± 4.5	9.3 ± 5.8	10.4 ± 7.3
*tpehgt_p003*	Dummy	9.0 ± 5.5	8.7 ± 5.6	8.6 ± 4.1	9.6 ± 5.5	9.5 ± 4.9
	Contraction	9.7 ± 7.1	8.5 ± 6.5	7.4 ± 4.7	10.0 ± 6.5	8.3 ± 7.2
*tpehgt_p004*	Dummy	8.5 ± 6.1	8.8 ± 6.1	7.6 ± 4.2	9.7 ± 5.3	8.2 ± 7.0
	Contraction	10.6 ± 8.5	9.3 ± 7.2	7.6 ± 4.4	9.5 ± 6.1	9.6 ± 6.9
*tpehgt_p005*	Dummy	8.7 ± 5.6	8.7 ± 5.5	8.5 ± 4.5	9.0 ± 4.9	9.4 ± 5.8
	Contraction	9.2 ± 6.4	8.5 ± 5.4	8.1 ± 4.3	9.4 ± 5.3	9.9 ± 6.0
*tpehgt_p006*	Dummy	10.3 ± 6.8	9.6 ± 6.2	8.7 ± 4.2	9.3 ± 5.6	9.7 ± 6.0
	Contraction	10.1 ± 6.9	9.3 ± 6.1	8.2 ± 4.6	9.9 ± 6.5	10.3 ± 6.6
*tpehgt_p007*	Dummy	9.9 ± 6.7	9.4 ± 6.5	8.7 ± 5.5	9.9 ± 5.9	10.4 ± 6.4
	Contraction	9.6 ± 7.4	9.6 ± 7.5	7.3 ± 4.8	10.2 ± 6.5	10.5 ± 7.6
*tpehgt_p008*	Dummy	9.8 ± 6.6	8.6 ± 5.8	7.6 ± 4.0	9.7 ± 5.7	8.9 ± 5.5
	Contraction	9.2 ± 6.3	8.4 ± 5.9	7.2 ± 4.2	9.8 ± 6.3	8.8 ± 6.1
*tpehgt_p009*	Dummy	9.6 ± 6.3	8.8 ± 5.6	8.3 ± 4.2	9.7 ± 5.4	9.8 ± 6.5
	Contraction	9.3 ± 6.1	9.0 ± 5.7	8.2 ± 4.4	9.9 ± 6.1	10.3 ± 7.1
*tpehgt_p010*	Dummy	8.5 ± 4.3	8.8 ± 4.6	8.5 ± 4.2	8.9 ± 4.6	9.9 ± 6.3
	Contraction	9.3 ± 5.4	9.2 ± 5.0	8.6 ± 4.5	9.4 ± 5.5	10.2 ± 6.2
*tpehgt_p011*	Dummy	9.1 ± 4.6	9.5 ± 5.0	8.5 ± 4.2	9.2 ± 4.7	9.2 ± 5.3
	Contraction	9.4 ± 5.4	10.0 ± 5.6	8.6 ± 5.0	9.6 ± 5.4	9.1 ± 6.3
*tpehgt_p012*	Dummy	9.2 ± 4.9	9.2 ± 4.8	9.5 ± 4.9	9.2 ± 4.7	9.5 ± 4.7
	Contraction	9.0 ± 5.3	9.3 ± 5.4	9.5 ± 5.3	8.6 ± 4.3	8.9 ± 4.8
*tpehgt_p013*	Dummy	9.2 ± 6.3	8.3 ± 5.2	8.1 ± 4.2	8.4 ± 5.2	9.1 ± 6.1
	Contraction	9.3 ± 6.8	8.7 ± 6.3	7.0 ± 4.5	6.0 ± 5.1	6.7 ± 7.9
*Aggregate average*	Dummy	9.2 ± 0.6	8.9 ± 0.4	8.4 ± 0.6	9.4 ± 0.4	9.5 ± 0.5
	Contraction	9.5 ± 0.4	9.0 ± 0.5	8.0 ± 0.7	9.3 ± 1.1	9.4 ± 1.1
				**$\bar{\mathrm{CV}}$ [cm/s]**	
**Term Records**	**Intervals**	**B0${}^{\prime }$**	**Bb**	**B1**	**B2**	**B3**
*tpehgt_t001*	Dummy	9.1 ± 5.7	9.2 ± 5.4	8.7 ± 4.6	9.5 ± 5.0	9.6 ± 4.9
	Contraction	9.7 ± 6.6	8.6 ± 5.3	8.6 ± 4.3	9.7 ± 4.8	9.3 ± 5.0
*tpehgt_t002*	Dummy	9.4 ± 5.4	9.1 ± 4.9	8.7 ± 4.3	9.1 ± 4.7	9.8 ± 5.6
	Contraction	9.3 ± 6.5	9.0 ± 5.9	7.7 ± 4.5	9.5 ± 5.0	9.9 ± 6.2
*tpehgt_t003*	Dummy	9.2 ± 5.6	9.1 ± 5.4	8.7 ± 4.3	9.6 ± 5.5	9.6 ± 5.4
	Contraction	9.0 ± 5.9	8.5 ± 5.7	7.9 ± 4.6	9.7 ± 6.0	9.6 ± 5.9
*tpehgt_t004*	Dummy	9.4 ± 6.6	7.8 ± 5.7	6.5 ± 4.3	9.7 ± 6.7	9.5 ± 6.7
	Contraction	10.2 ± 6.6	9.6 ± 6.2	8.1 ± 4.6	9.7 ± 5.2	10.3 ± 6.5
*tpehgt_t005*	Dummy	8.9 ± 4.6	8.6 ± 4.5	8.9 ± 4.4	9.6 ± 4.7	9.1 ± 4.6
	Contraction	9.2 ± 5.3	8.8 ± 4.5	8.8 ± 4.9	8.3 ± 4.2	9.3 ± 5.3
*tpehgt_t006*	Dummy	8.7 ± 5.9	8.8 ± 5.7	8.8 ± 4.8	9.1 ± 4.8	9.8 ± 5.6
	Contraction	9.0 ± 5.9	8.9 ± 5.6	8.4 ± 4.9	9.5 ± 5.4	9.6 ± 5.6
*tpehgt_t007*	Dummy	9.0 ± 4.9	9.1 ± 4.6	8.5 ± 4.1	9.4 ± 4.9	9.7 ± 5.9
	Contraction	8.8 ± 5.3	8.8 ± 4.8	8.2 ± 4.7	9.0 ± 5.1	10.0 ± 6.7
*tpehgt_t008*	Dummy	8.9 ± 5.1	8.8 ± 4.7	8.7 ± 4.4	9.3 ± 4.9	9.6 ± 5.7
	Contraction	9.1 ± 5.3	9.0 ± 5.1	8.6 ± 4.6	9.4 ± 4.8	9.2 ± 5.3
*tpehgt_t009*	Dummy	9.5 ± 6.4	8.8 ± 5.6	8.3 ± 4.7	9.6 ± 5.7	10.2 ± 7.6
	Contraction	9.2 ± 6.6	8.7 ± 5.9	8.0 ± 4.8	9.8 ± 5.9	10.4 ± 7.8
*tpehgt_t010*	Dummy	9.0 ± 5.0	8.9 ± 4.9	8.8 ± 4.7	9.9 ± 5.2	10.0 ± 6.4
	Contraction	9.3 ± 6.0	8.8 ± 5.7	8.0 ± 4.4	9.8 ± 5.2	10.0 ± 6.9
*tpehgt_t011*	Dummy	9.2 ± 5.8	8.3 ± 5.1	7.4 ± 3.4	8.6 ± 6.3	8.5 ± 7.4
	Contraction	9.3 ± 5.7	8.7 ± 5.5	7.4 ± 3.6	8.9 ± 6.1	8.4 ± 7.1
*tpehgt_t012*	Dummy	8.7 ± 5.7	8.9 ± 5.3	9.4 ± 4.7	9.4 ± 5.8	9.5 ± 5.6
	Contraction	8.0 ± 4.4	8.6 ± 5.0	9.8 ± 4.8	9.9 ± 6.1	9.6 ± 6.1
*tpehgt_t013*	Dummy	8.5 ± 5.2	8.9 ± 5.2	8.3 ± 4.3	9.3 ± 4.7	9.4 ± 5.4
	Contraction	9.3 ± 6.2	9.1 ± 5.7	8.5 ± 4.6	10.0 ± 5.1	9.9 ± 6.1
*Aggregate average*	Dummy	9.0 ± 0.3	8.8 ± 0.4	8.4 ± 0.7	9.4 ± 0.3	9.6 ± 0.4
	Contraction	9.2 ± 0.5	8.9 ± 0.3	8.3 ± 0.6	9.5 ± 0.5	9.7 ± 0.5

**Table A2:** Average conduction velocity amplitudes, $\bar{CV}$, as estimated over the entire preterm and term EHG records of the TPEHGT DS. For the time courses of the conduction velocity amplitudes, CV, of the gray-shaded records, see Figure A3 and  Figure A4.

			$\bar{\mathrm{CV}}$ [cm/s]		
Preterm Records	B0${}^{\prime }$	Bb	B1	B2	B3
*tpehgt_p001*	9.1 ± 5.2	8.8 ± 4.9	9.1 ± 4.7	9.4 ± 5.2	9.5 ± 5.8
*tpehgt_p002*	9.5 ± 6.4	8.8 ± 5.8	7.9 ± 4.5	9.4 ± 5.8	9.9 ± 6.6
*tpehgt_p003*	9.3 ± 6.1	8.9 ± 5.8	8.1 ± 4.5	9.7 ± 5.8	9.1 ± 5.7
*tpehgt_p004*	9.3 ± 7.1	8.7 ± 6.4	7.5 ± 4.4	9.2 ± 5.9	8.8 ± 7.4
*tpehgt_p005*	8.9 ± 6.2	8.7 ± 5.9	8.3 ± 4.8	9.2 ± 4.9	9.9 ± 6.2
*tpehgt_p006*	10.4 ± 7.1	9.6 ± 6.4	8.5 ± 4.5	9.6 ± 6.1	10.0 ± 6.3
*tpehgt_p007*	9.7 ± 6.7	9.2 ± 6.4	8.0 ± 5.1	9.8 ± 6.2	10.4 ± 7.0
*tpehgt_p008*	9.6 ± 6.3	8.7 ± 5.8	7.6 ± 4.1	9.6 ± 5.8	9.0 ± 5.6
*tpehgt_p009*	9.4 ± 6.2	8.9 ± 5.7	8.3 ± 4.3	9.8 ± 5.7	10.1 ± 6.8
*tpehgt_p010*	8.9 ± 4.9	9.0 ± 4.9	8.5 ± 4.3	9.2 ± 5.0	10.0 ± 6.2
*tpehgt_p011*	9.0 ± 4.8	9.1 ± 4.8	8.7 ± 4.5	9.6 ± 5.2	9.0 ± 5.5
*tpehgt_p012*	9.3 ± 5.0	9.3 ± 4.8	9.3 ± 4.7	9.2 ± 4.7	9.4 ± 4.9
*tpehgt_p013*	9.2 ± 6.1	8.8 ± 5.8	7.9 ± 4.5	7.2 ± 5.0	8.5 ± 7.0
*Aggregate average*	9.4 ± 0.4	9.0 ± 0.3	8.3 ± 0.5	9.3 ± 0.7	9.5 ± 0.6
			**$\bar{\mathrm{CV}}$ [cm/s]**	
**Term Records**	**B0${}^{\prime }$**	**Bb**	**B1**	**B2**	**B3**
*tpehgt_t001*	9.1 ± 5.7	9.0 ± 5.2	8.9 ± 4.5	9.5 ± 4.9	9.2 ± 4.6
*tpehgt_t002*	9.4 ± 6.0	8.8 ± 5.4	8.1 ± 4.5	9.3 ± 4.8	9.8 ± 5.7
*tpehgt_t003*	9.2 ± 5.9	8.8 ± 5.5	8.3 ± 4.4	9.8 ± 5.7	9.6 ± 5.7
*tpehgt_t004*	9.4 ± 6.2	8.9 ± 6.0	8.0 ± 4.5	9.6 ± 5.7	10.3 ± 6.6
*tpehgt_t005*	9.0 ± 5.5	8.8 ± 5.1	8.8 ± 4.6	9.1 ± 4.6	9.4 ± 5.3
*tpehgt_t006*	8.8 ± 5.9	8.7 ± 5.5	8.5 ± 4.7	9.3 ± 5.1	9.7 ± 5.5
*tpehgt_t007*	9.1 ± 5.1	8.9 ± 4.7	8.6 ± 4.4	9.2 ± 4.7	9.8 ± 6.1
*tpehgt_t008*	9.1 ± 5.3	8.9 ± 4.9	8.6 ± 4.4	9.3 ± 4.7	9.4 ± 5.6
*tpehgt_t009*	9.4 ± 6.5	8.7 ± 5.7	8.2 ± 4.7	9.7 ± 5.8	10.2 ± 7.6
*tpehgt_t010*	9.0 ± 5.2	9.0 ± 5.2	8.5 ± 4.5	9.8 ± 5.3	10.0 ± 6.4
*tpehgt_t011*	9.2 ± 5.6	8.6 ± 5.3	7.5 ± 3.5	8.8 ± 6.1	8.6 ± 7.2
*tpehgt_t012*	8.4 ± 5.2	8.7 ± 5.3	9.3 ± 4.7	9.5 ± 5.8	9.4 ± 5.7
*tpehgt_t013*	8.9 ± 5.6	8.9 ± 5.3	8.4 ± 4.3	9.5 ± 4.8	9.6 ± 5.7
*Aggregate average*	9.1 ± 0.3	8.8 ± 0.1	8.4 ± 0.5	9.4 ± 0.3	9.6 ± 0.4
